# Binding Affinity Determines the Success of Endogenous Cu^II^-NTA Spin Labeling for In-Cell Electron Paramagnetic Resonance Distance Measurements

**DOI:** 10.1021/acs.jpclett.6c01384

**Published:** 2026-06-05

**Authors:** Yannik Limbach, Katrin Ackermann, Olav Schiemann, Bela E. Bode

**Affiliations:** † EaStCHEM School of Chemistry, Biomedical Sciences Research Complex and Centre of Magnetic Resonance, 150654 University of St. Andrews, North Haugh, St Andrews, KY16 9ST Scotland, U.K.; ‡ Clausius-Institute for Physical and Theoretical Chemistry, 9374 University of Bonn, Wegelerstr. 12, 53115 Bonn, Germany

## Abstract

In-cell electron paramagnetic resonance (EPR) spectroscopy requires robust spin-labeling strategies compatible with cellular conditions. Cu^II^-NTA coordination to genetically engineered double-histidine (dHis) motifs has shown promise for endogenous labeling in *Escherichia coli*. Here, we evaluated the effect of varying Cu^II^-NTA affinity on the success of these experiments. Relaxation induced dipolar modulation enhancement (RIDME)-based titrations revealed dissociation constants (*K*_d_) in the 10^–6^ and 10^–8^ range for two different β-sheet *i* and *i*+2 dHis sites. In-cell EPR spectra demonstrated that the persistence of the Cu^II^ EPR signal correlates with these binding affinities. The success of in-cell pulsed electron–electron double resonance (PELDOR) involving both β-sheet sites and a high-affinity α-helical site depended on the site; the higher-affinity site yielded analyzable results, whereas the lower-affinity site did not. These results highlight the critical importance of low *K*_d_ binding sites for reliable in-cell distance measurements with endogenous Cu^II^-NTA labeling and the substantial sensitivity gain offered by RIDME experiments.

## Graphical abstract

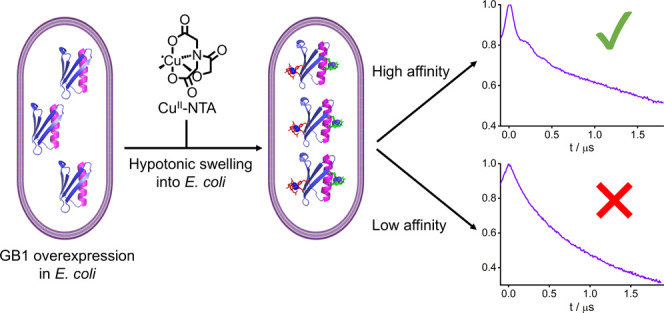

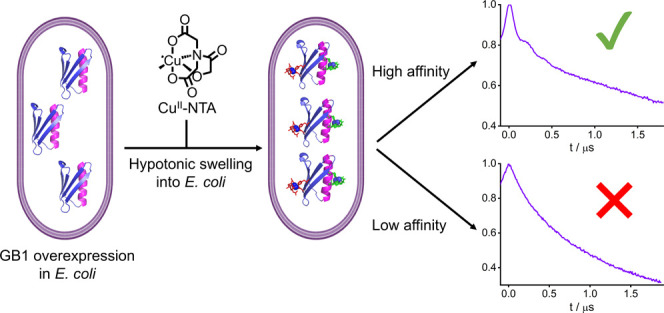

Understanding the structure and conformational dynamics of proteins inside living cells is a central goal of modern structural biology. While high-resolution techniques such as X-ray crystallography and cryoelectron microscopy provide detailed structural information, they typically require purified samples and non-native conditions. In contrast, pulsed dipolar electron paramagnetic resonance (EPR) spectroscopy (PDS) enables nanometer-scale distance measurements^1^ under frozen-solution conditions and is uniquely suited to investigate biomolecular structures in complex environments, including intact cells.^2^ Among PDS methods, pulsed electron–electron double resonance (PELDOR/DEER)^3,4^ and relaxation-induced dipolar modulation enhancement (RIDME)^5,6^ have become powerful tools for extracting distance distributions between paramagnetic centers grafted onto biomolecules.

A key prerequisite for the application of PDS methods in the cellular context is the availability of spin-labeling strategies that are both site-specific and yield sufficiently persistent paramagnetic centers in the reducing intracellular environment.^7^ Traditional cysteine-reactive nitroxide labels often suffer from rapid reduction in cells, severely limiting their applicability.^8,9^ Further advances in spin-label chemistry, including the development of sterically shielded nitroxides,^10–13^ gadolinium^III^-,^14–17^ and trityl-based^18–22^ spin labels, have markedly enhanced intracellular lifetimes and broadened the scope of in-cell PDS distance measurements.

In addition to the development of spin labels tailored to the reductive environment within cells, new methods to deliver biomolecules of interest into different organisms were developed. In early studies, oocytes from *Xenopus laevis* were transfected *via* microinjection.^23^ −^24,25,26^ To make a broader range of biological systems accessible, a more advanced delivery *via* electroporation has been established.^2^ ,^14^ However, if studies are performed on mammalian cells,^2,17,27^ these require comparably long recovery times after electroporation, which significantly affects the resulting data quality. In contrast, promising results were recently achieved through electroporation of *Escherichia coli* ^28,29^ bacteria since their comparably short recovery period appears beneficial for data quality. Nevertheless, techniques that rely on protein delivery have drawbacks, such as the time-consuming preparation of spin-labeled protein systems *in vitro*. To overcome these issues, some effort has been directed toward developing endogenous spin labeling approaches that enable labeling of a target protein within the cell of interest. Most of those techniques rely on the incorporation of noncanonical amino acids,^30–32^ which usually lowers the expression of the studied proteins significantly, and the removal of excess labeling reagent might prove challenging. More recently, in-cell labeling was achieved by introducing Cu^II^-nitrilotriacetic acid (NTA) into bacteria expressing proteins with *cis* double-histidine (dHis) mutations that display high affinity for this complex.^33,34^ This approach has been used extensively for *in vitro* studies in the literature,^35–38^ and the introduction of dHis sites into α-helices and β-sheets is an established tool for spin-labeling *via* complexation of Cu^II^-NTA onto proteins.

Despite these promising developments, the general applicability of Cu^II^-NTA labeling in cells remains insufficiently understood. Because Cu^II^ coordination to dHis motifs is governed by a dynamic equilibrium,^38^ the dissociation constant (*K*_d_) of the binding site is expected to play a critical role in determining the fraction of successfully labeled protein and, consequently, the modulation depth and sensitivity of PDS measurements. Saturation of the binding site is not easily possible within cells since the Cu^II^-NTA concentration needs to be limited to maintain cell viability.^33^ Variations in histidine positioning can substantially alter binding affinity,^39–41^ yet a systematic evaluation of how this affects in-cell measurements is lacking. It remains unclear to what extent weaker binding sites compromise the persistence of the Cu^II^-NTA dHis complex which might lead to reduction under intracellular conditions and thereby limit structural investigations. In this work, we systematically investigate the influence of the dHis binding-site affinity on EPR spectra and RIDME results.

We proceeded along previously published results described by Hunter *et al*.^33^ with minor modifications (Figure 1a and SI, section 1.2). Notably, we used a different GB1 (*Streptococcus sp.* group G protein G, B1 domain) mutant, namely, GB1 6H8H/28H32H (Figure 1b), instead of GB1 15H17H/28H32H. These GB1 mutants were excessively studied in previous work to understand the dHis Cu^II^-NTA binding affinity and competition with adventitious metal ions.^38,39,42,43^ Since the protein system was not changed and only the position of one double histidine was altered, we did not anticipate significant differences in the outcome. However, we could not generate a PELDOR measurement (parameters, SI section 1.5.3) with a visible dipolar oscillation (Figure 1c). The resulting time trace could be described by an exponential decay and, thus, could not be used to extract a distance distribution. This result highlighted the question of the general applicability of this approach. For general applicability, transfer to other systems and binding sites is crucial, and an understanding of the minimum requirements is necessary. We hypothesized that the higher *K*_d_ of the 6H8H binding site caused the failure of the in-cell distance measurements, which required further insight into the sites involved to be obtained.

**Figure 1:**
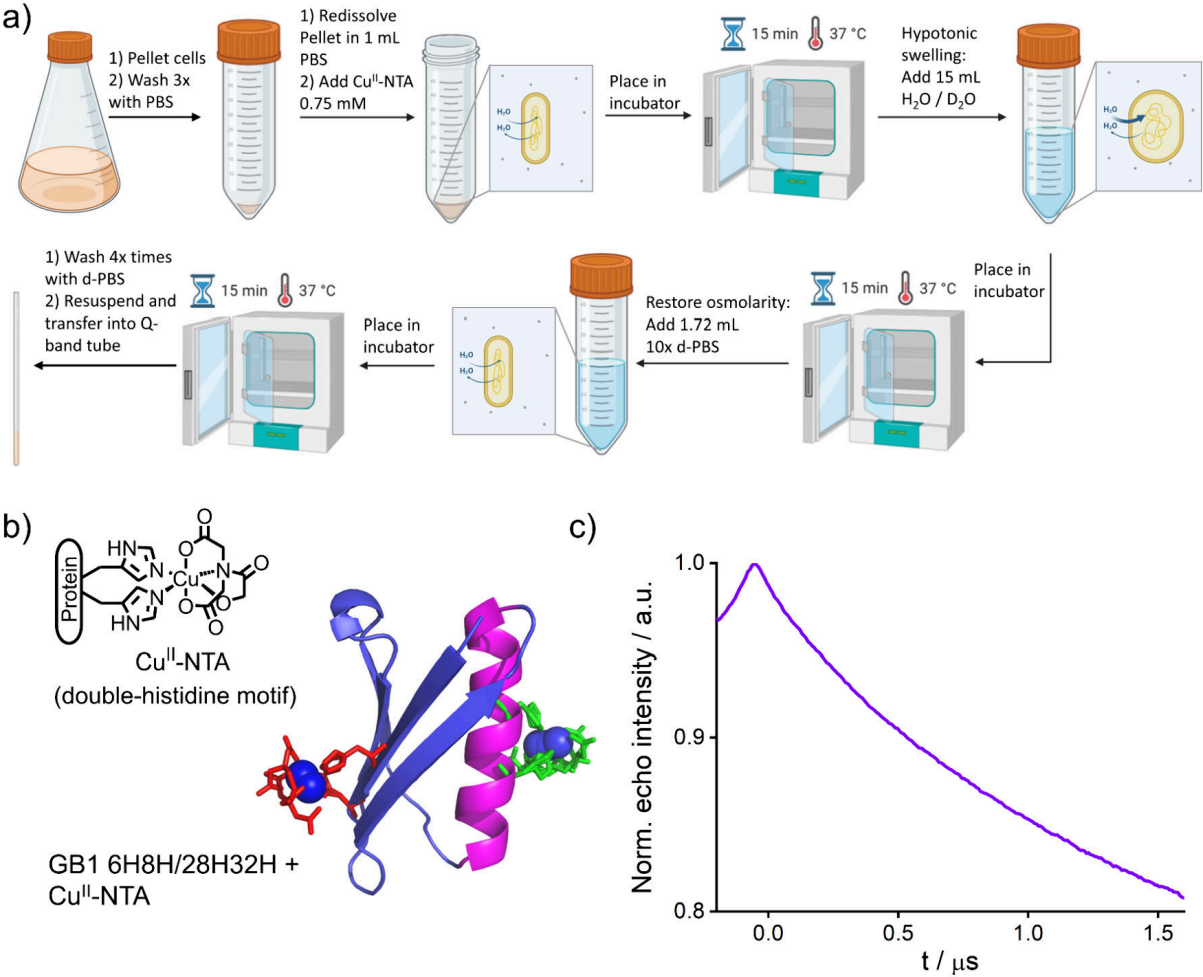
Overview of the procedure for in-cell sample preparation and results for the GB1 6H8H/28H32H mutantin *E. coli*. a) Schematic of the workflow carried out, created with Biorender.com.^44^ b) Crystal structure of GB1 (PDB: 4WH4)^38^ with the rotamer cloud of the Cu^II^-NTA labeled 6H8H and 28H32H dHis motifs, generated in ChiLife.^45–47^ c) PELDOR measurement, carried out at Q-band and at 18 K.

The dissociation constants *K*_d_ of the binding sites were determined to investigate the stability of the two formed dHis Cu^II^-NTA complexes. Therefore, pseudo titration series were carried out, as described earlier.^39,48,49^ To investigate the binding sites independently, mixed mutants with only one dHis site and a methanethiosulfonate spin label (MTSL, R1) replacing the other site—GB1 15H17H/28R1 and 6H8H/28R1—were designed (Figure 2a,b), expressed, purified, and characterized (SI, section 2.1 and 2.2) as described earlier.^39^ Correct folding of those and the later used mutants was confirmed in previous studies *via* circular dichroism (CD)^38^ ,^39^ ,^43^ and an X-ray structure.^38^ Since the determination of the *K*_d_ becomes more accurate when the protein concentration is in a similar range,^40^ the 15H17H mutant was measured at 0.5 μM concentration because a *K*_d_ of 10^–7^ to 10^–8^ was expected, and the 6H8H mutant was measured at 5 μM, expecting a *K*_d_ of 10^–6^ to 10^–7^. The added Cu^II^-NTA concentrations were calculated such that 20, 40, 60, 80, and 95% binding site occupation should be achieved based on estimated *K*_d_ values as described in the Supporting Information (SI, section 1.3).

**Figure 2:**
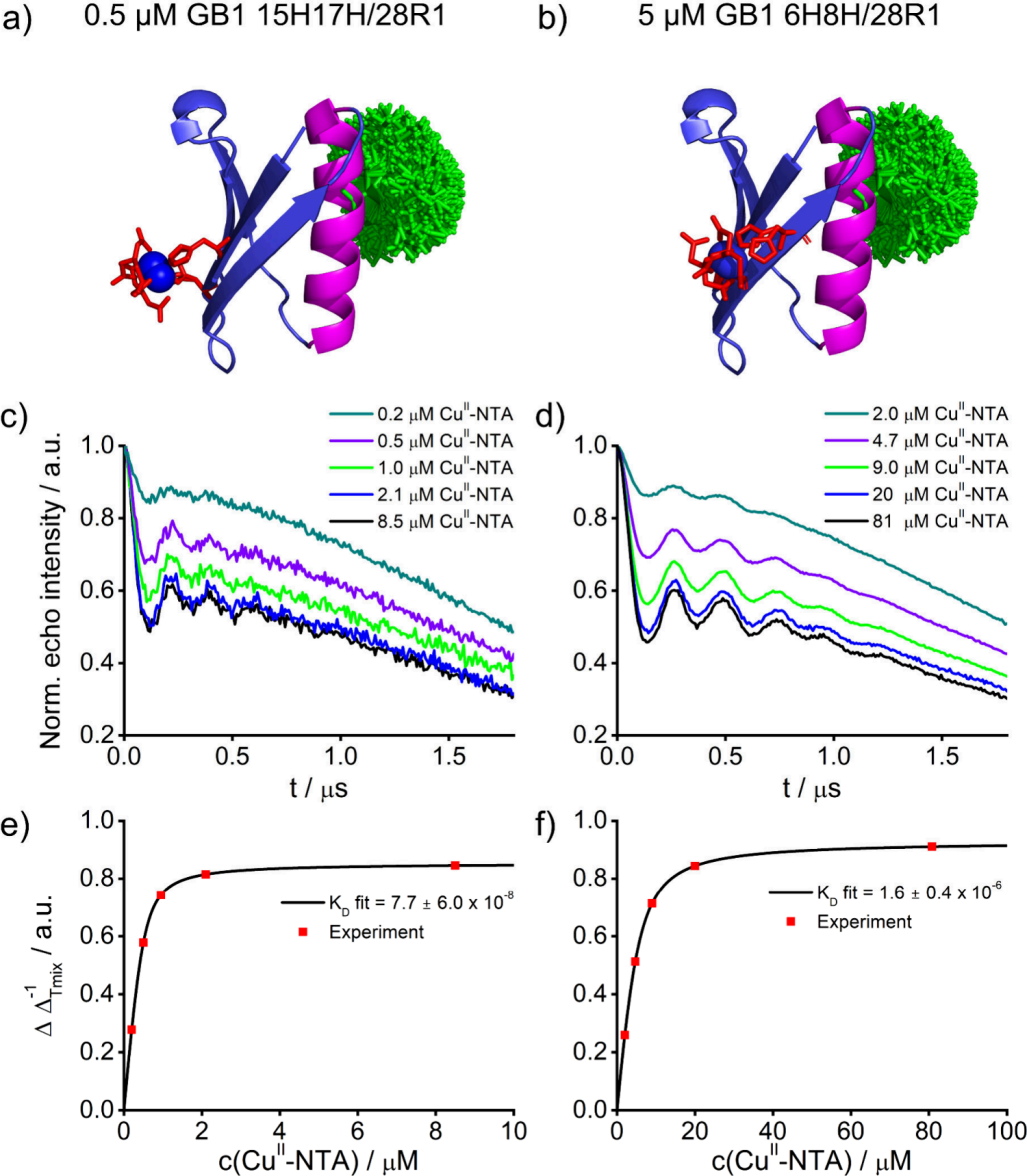
Dissociation constant *K*_d_ evaluation of different GB1 dHis sites. a) A crystal structure of GB1 (PDB: 4WH4) with the rotamer cloud of the MTSL labeled 28C mutant and the Cu^II^-NTA labeled 15H17H dHis motif, generated in ChiLife.^45,50^ b) As in a) for the GB1 6H8H/28R1 mutant. c) Deconvoluted RIDME measurements on the maximum of the MTSL echo detected field sweep spectrum (EDFS) of 0.5 μM GB1 15H17H/28R1 with increasing Cu^II^-NTA concentrations. d) Deconvoluted RIDME measurements on the maximum of the MTSL EDFS spectrum of 5 μM GB1 6H8H/28R1 with increasing Cu^II^-NTA concentrations. e, f) Scaled experimental modulation depths Δ/Δ_Tmix_ of the RIDME measurements in (c, d) (red dots) as a function of the Cu^II^-NTA concentration and the fitted binding isotherm (black).

The resulting deconvoluted RIDME measurements (Figure 2c,d, parameters are described in SI, section 1.5.4) show the expected trend featuring decreasing modulation depths and increasing *T*_1_ relaxation times (SI, section 2.3) when reducing the Cu^II^-NTA concentration. The fitting of binding isotherms to the modulation depth data in Figure 2e,f resulted in *K*_d_ values of 7.7 ± 6.0 × 10^–8^ in the case of the 15H17H site and 1.6 ± 0.4 × 10^–6^ for the 6H8H site. In a previous study the *K*_d_ of the site 28H32H was determined to be 1.4 × 10^–7^.^39^ The isotherm fitting was carried out as previously described^39^ and as outlined in the Supporting Information (SI, section 2.3). This result highlights that the 6H8H/28H32H mutant features a more than 1 order of magnitude lower affinity at the 6H8H binding site which is hypothesized to be the crucial difference to the experiment carried out by Hunter *et al*.^33^ To further assess the influence of the *in vitro* determined *K*_d_ on the successful application of in-cell distance measurements, the binding sites were investigated independently from each other within *E. coli.*

For studying the individual binding sites within *E. coli*, GB1 6H8H, 15H17H, and 28H32H encoding plasmids were prepared (SI, section 1.1). The previously described procedure was carried out with all three of these mutants. Since the designed mutants only contain one Cu^II^-NTA binding site, investigations with PDS were not considered. Instead, CW-EPR spectra of the respective samples were recorded (Figure 3, parameters are described in SI, section 1.4). To analyze the results, two reference spectra were selected. Representing the successfully formed complex, an *in vitro* sample of 250 μM GB1 15H17H/28H32H with Cu^II^-NTA was used (Figure 3a), described as component 1. The opposite example was created by swelling of Cu^II^-NTA into *E. coli* that did not express GB1, which will be described as component 2 (Figure 3b). The two spectra differed significantly in their intensity and component 2 showed additional hyperfine coupling on *g*_⊥_. On the parallel component of the spectra *g*_∥_-values decreased and the hyperfine splitting *A*_∥_ increased. A high fraction of component 1 suggests successful binding of Cu^II^-NTA to the respective site, whereas a low fraction of component 1 represents Cu^II^-NTA that is present elsewhere in the bacterium.

**Figure 3:**
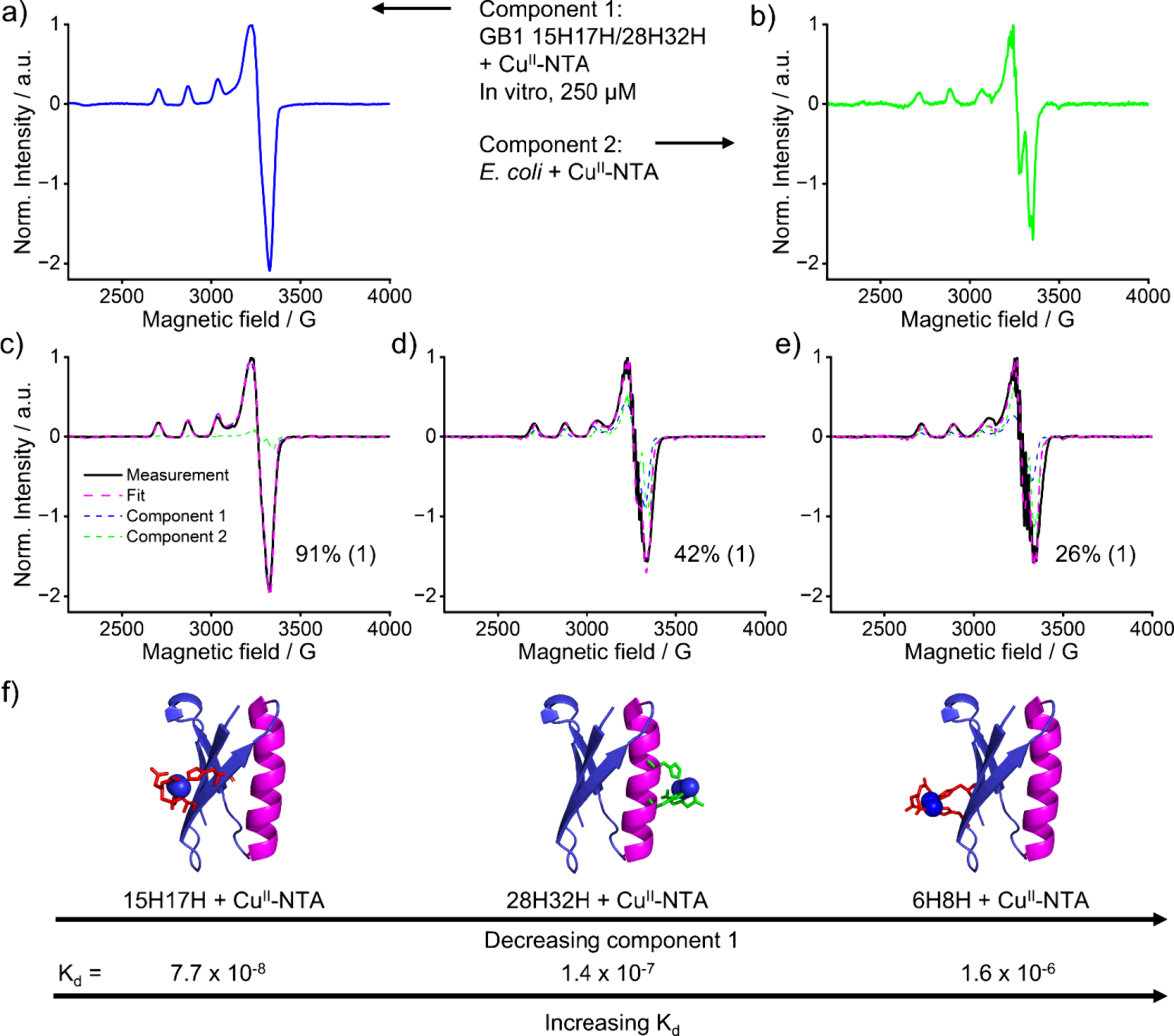
Investigation of different GB1 dHis motifs in *E. coli* *via* CW-EPR spectroscopy measured at 120 K. a) Bound state *in vitro* reference sample, 250 μM GB1 15H17H/28H32H with 1:2 Cu^II^-NTA (blue) used as component 1 for fitting. b) Cu^II^-NTA background sample, nonexpressing *E. coli* cells swelled with Cu^II^-NTA used as component 2 for fitting. GB1 mutant c) 15H17H, d) 28H32H and e) 6H8H overexpressed in *E. coli* swelled with Cu^II^-NTA. Depicted is the measurement (black), the fit (magenta, dash), the fraction of component 1 (blue, dash), and the fraction of component 2 (green, dash). f) The different dHis sites labeled with Cu^II^-NTA in ChiLife, corresponding to the measurements above. Trends for component 1 and the *K*_d_ are depicted.

The simplest way to fit the individual components to the data is by linearly combining the two individual spectra (SI, section 1.4.3). This was conducted for three binding sites. The highest fraction of component 1 was found in the 15H17H sample (Figure 3c). About 91% of the measurable Cu^II^-NTA seemed to be in the bound state. The fraction of component 1 decreased from 42% to 26% for the 28H32H (Figure 3d) and 6H8H (Figure 3e) mutants, respectively. This trend highlights the influence of the binding site and indicates that the 6H8H/28H32H dHis combination will only yield a low fraction of doubly labeled protein, which could explain the unsuccessful distance measurements (Figure S14). In contrast, the 15H17H/28H32H mutant contained a higher fraction of doubly labeled protein (Figure S13) because of the 15H17H binding site, which aligns well with the reported distance measurements on this mutant.

Looking at the respective binding site, their *K*_d_ values increased with decreasing component 1 (Figure 3f). Therefore, it is highly likely that for this experiment to produce useful distance measurements dHis Cu^II^-NTA complexes with very low *K*_d_’s are needed. α-Helical sites have been suggested to provide higher affinities than β-sheet sites.^43^ However, the 15H17H β-sheet and 28H32H α-helix mutants have similar affinity pointing to a more complicated relationship between binding motif and *K*_d_.

This was further investigated by looking at the resulting Cu^II^-NTA signal when incubating the swelled cells for increasing time frames (SI, section 2.5.1), effectively suggesting that even complexes containing the 15H17H site disassemble over time, and the released Cu^II^-NTA is then subsequently reduced. This manifests in the CW-EPR data as a reduction in signal intensity (Figure S11c), meaning that for Cu^II^-NTA to be persistent within the cellular environment, it needs to be bound to a dHis binding site. The lower the binding site *K*_d_, the more stable and longer-lived the resulting Cu^II^-NTA dHis complex.

Other factors such as the influence of different expression times were tested as well (SI, Section 2.5.2). All of the previously presented samples were prepared after overnight expression. However, the results from this experiment (SI, section 2.5.2) were less conclusive; only the sample with overnight expression resulted in a sample allowing for distance extraction. The other samples did not show the expected CW-EPR intensities. The causes of this trend are not obvious and require further investigations.

Having established that the *K*_d_ of the dHis motif is crucial for successful application of PDS methods, we chose the GB1 15H17H/28H32H mutant (Figure 4a) for tests to improve the sensitivity of in-cell PDS measurements. As previously described by Hunter *et al*.,^33^ this mutant should allow for distance extraction in the cellular environment. To investigate whether the sensitivity could be improved over the published procedure, we decided to perform the swelling and all subsequent washing steps in D_2_O and a deuterated buffer. This has the advantage that some degree of deuteration is achievable,^51^ prolonging the phase memory time *T*_m_ (SI, Section 2.6) and subsequently allowing for the application of RIDME measurements. The treatment of the bacteria with deuterated solvents did not significantly influence cell viability (Figure S11b). Additionally, negative controls were prepared (Figure S10), excluding leakage of expressed proteins and the presence of distances due to unspecific binding of Cu^II^-NTA. Using RIDME instead of PELDOR for measurements involving Cu^II^ is often beneficial for the sensitivity of the measurement^37,39,52^ because RIDME can exploit spontaneous spin flips independent of the current field positions, whereas the pump pulse in PELDOR can only flip a fraction of the Cu^II^ spins even when applying broadband pulses. The two different measurements were carried out at three different field positions (Figure 4b)^37,53^ to account for orientation selection. RIDME and PELODR experiments were set up as described in the Supporting Information (sections 1.5.2 and 1.5.3).

**Figure 4:**
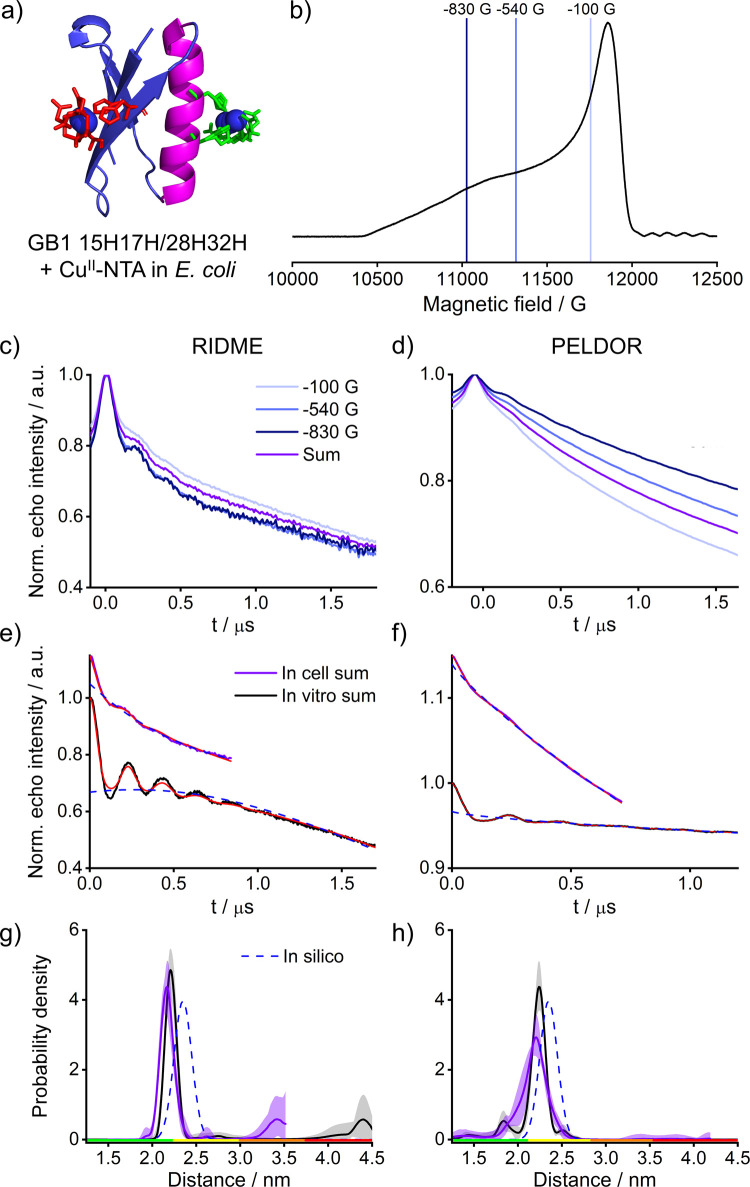
RIDME and PELDOR measurements on the GB1 mutant 15H17H/28H32H in *E. coli* swelled with Cu^II^-NTA. a) Representation of the crystal structure (PDB: 4HW4), with the two dHis sites 15H17H and 28H32H labeled with Cu^II^-NTA in ChiLife. b) EDFS spectrum of the sample measured at 18 K with the respective measurement positions. c) RIDME measurements at 30 K in the color code as in (b) with the summed trace (violet). d) PELDOR measurements at 18 K in the color code as in (b). e) The summed RIDME measurements of the in-cell sample (violet, same as in (c)) and *in vitro* reference sample of GB1 15H17H/28H32H (black) analyzed with the CDA2.0. The resulting fit is depicted in red and the background as blue dashed lines. f) The summed PELDOR measurements of the samples in (e). g) Calculated distance distributions resulting from the fits in (e). Additionally, an *in silico* prediction (blue, dash) generated with ChiLife as well as a color bar from green to red depicting the reliability of the distance distribution is shown. h) Calculated distance distributions resulting from the fits in (f), in the same color code as in (g).

The resulting RIDME measurements (Figure 4c) contain a visible dipolar oscillation and a significantly larger modulation depth (∼10%) in comparison to the PELDOR measurements (Figure 4d) (∼1.3%). The individual traces were weighed by the echo detected field sweep (EDFS) intensities at the respective field positions and summed for analysis with the comparative DEER analyzer (CDA2.0).^54,55^ Comparing the resulting fits of the summed RIDME trace with an *in vitro* reference (Figure 4e), it is visible that the modulation depth is lower and the background decay steeper. This behavior might originate from incomplete labeling in-cell and an inferior deuteration degree within the cells compared to the *in vitro* sample. This trend was observed for the PELDOR measurement as well (Figure 4f). The resulting distance distributions align very well with the reference measurements for both the PELDOR and RIDME method. A shift of the average distance to shorter values by 1.7 Å (RIDME) and 1.6 Å (PELDOR) is visible (Figure 4g,h) in comparison to the *in silico* prediction. This effect was observed in the literature for the same GB1 mutant and was attributed to the prediction uncertainty of about 1 Å which might be further increased by the chosen crystal structure for modeling.^46^ For the PELDOR distributions (Figure 4h) a broadening of the in-cell measurement could be observed, which might be attributed to the lower modulation depth. Furthermore, to validate the resulting distance distributions biological repeats were prepared (SI, section 2.7). The RIDME measurements resulted in very similar distance distributions and average distances; some deviations were encountered when looking at the modulation depths and the background decays, leading to higher uncertainties for the second repeat (SI, section 2.7). However, this might be explained by the different expression levels and the fraction of doubly labeled GB1 which might vary between experiments.

Both methods resulted in comparable average distances and standard deviations (Table 1). When comparing the signal-to-noise ratio (SNR) weighted by the square root of the acquisition time (SNR_t_) (Table 1), the benefits of RIDME became evident. The increase in SNR_t_ was about 5-fold, considering the loss in SNR due to deconvolution of the RIDME traces. Furthermore, if a conventional PELDOR set up with rectangular pulses would have been used the resulting sensitivity gain would be even more pronounced in favor of RIDME.

**Table 1:** Acquisition Time (*t*), SNR, Weighted SNR_t_ Values, Average Distance (<*r*>) and Standard Deviation of the Average (σ_r_) of the in-Cell Measurements Presented in Figure 4

	*t*/h	SNR	SNR_t_/h^‑^1/2	<*r*>/nm	σ_r_/nm
RIDME sum	9.829	48.36	15.42	2.18	0.10
PELDOR sum	18.01	13.54	3.191	2.19	0.09

In summary, this work demonstrates that the success of endogenous Cu^II^-NTA spin labeling for in-cell PDS critically depends on the binding affinity of the engineered double-histidine motif. By systematically comparing GB1 mutants, we show that low-affinity sites such as 6H8H (*K*_d_ 10^–6^) lead to insufficient complex stability and a reduced fraction of doubly labeled protein in *E. coli*, ultimately preventing reliable distance measurements, whereas high-affinity sites such as 15H17H and 28H32H (*K*_d_ 10^–7^ to 10^–8^) enable robust in-cell PDS experiments. The correlation between *K*_d_ values determined by RIDME titrations and the fraction of bound Cu^II^-NTA observed by CW-EPR in cells highlights the central role of tight binding in preserving the complex in-cell. Furthermore, the combination of high-affinity motifs with partial deuteration allows RIDME measurements with substantially enhanced sensitivity compared to PELDOR while yielding consistent distance distributions. Further investigations are needed to study the influence of expression levels and the state of the bacteria while performing the swelling. To make this methodology useful for structural biology, predictors for binding site *K*_d_ need to be developed. Very recently a variety of different binding motifs of Cu^II^-NTA was described *via* X-ray crystallography,^41^ which might explain the high variability between binding site *K*_d_’s. The presence of experimental structures of binding motifs might be used to guide the development of computational tools to predict the stability of different dHis motifs. Additionally, the applicability to different protein systems and other cell types (e.g., mammalian cells) remains to be established. If successful, these methodologies might provide very powerful structural information on biomolecules directly within their native environment, without the need to purify the studied systems, significantly reducing the preparative effort.

## Supplementary Material



## Data Availability

The research data underpinning this publication are accessible at 10.17630/93fe2a7b-9357-470a-bd90-57917a1d5a3e.^56^

## Notes

Detailed experimental procedures, purification and characterization of GB1 mutantss, IR data for titration series, negative controls, expression and incubation time tests, CW data of additional GB1 in-cell samples, influence of deuteration (PDF)
