# Supplementary material for: Binding Affinity Determines the Success of Endogenous CuII-NTA Spin Labeling for In-Cell Electron Paramagnetic Resonance Distance Measurements
Source: J Phys Chem Lett. 2026 Jun 5;17(24):6736–44. doi: 10.1021/acs.jpclett.6c01384 (PMC13288622; doi:10.1021/acs.jpclett.6c01384)
Supplement: Supplementary file 1 [file jz6c01384_si_001.pdf]

Supporting Information

Binding Affinity Determines the Success of Endogenous Cu^{II}–NTA Spin Labeling for In-Cell EPR Distance Measurements

Yannik Limbach,[1,2] Katrin Ackermann,[1] Olav Schiemann,[2] Bela E. Bode[1]**

[1]EaStCHEM School of Chemistry, Biomedical Sciences Research Complex and Centre of Magnetic Resonance, University of St. Andrews, North Haugh, St Andrews KY16 9ST, Scotland, UK

[2]Clausius-Institute for Physical and Theoretical Chemistry, University of Bonn, Wegelerstr. 12, 53115 Bonn, Germany

AUTHOR INFORMATION

Corresponding Authors

*beb2@st-andrews.ac.uk; *schiemann@pc.uni-bonn.de

Table of Contents

1. Methods 3
   1.1 Mutant design, protein expression, purification and spin labeling 3
   1.2 In-cell sample preparation 3
   1.3 EPR sample preparation of *in vitro* samples and titration series 4
   1.4 CW-EPR experiments 5
   1.5 Pulse EPR experiments 7
   1.6 Mass spectrometry 10
2. Results and discussion 10
   2.1 Protein purification 10
   2.2 MTSL labeling 11
   2.3 Pseudo titration series, additional information 13
   2.4 Negative control samples 15
   2.5 Additional tests 16
   2.6 PELDOR and RIDME comparison 20
   2.7 Biological repeats RIDME measurements on GB1 15H17H/28H32H 22
3. Literature 23

1. Methods

1.1 Mutant design, protein expression, purification and spin labeling

For this study, the B1 domain of group G streptococcal protein G (GB1) from *Streptococcus sp.* was used. Specifically, the 6H8H/28H32H and 15H17H/28H32H mutants were used for in-cell PDS measurements. The 15H17H/28C and 6H8H/28C mutants were used for pseudo titrations and additionally the 28H32H and 6H8H for the in-cell CW measurements. The 15H17H/28C and 15H17H/28H32H mutants were newly prepared for this study, whereas the remaining mutants were described in previous studies.[1–4]

The mutants 15H17H/28C, 6H8H/28C and 15H17H/28H32H were expressed and purified as described earlier.[2,4] The 15H17H/28C and 6H8H/28C mutants were labelled with (1-Oxyl-2,2,5,5-tetramethyl-3-pyrroline-3-methyl)methanethiosulfonate (MTSL, Santa Cruz Biotechnology) under previously published conditions.[5] Since the 28C mutants are very prone to form disulfide bonds, reduction was carried out with increased amounts of DTT (140 x excess), in a prolonged reduction step overnight at 4 °C. Mass spectrometry has been conducted to confirm quantitative labeling. The MTSL labelled/purified samples were freeze dried and later redissolved in equal amounts of D_2O for preparation of reference and titration samples.

1.2 In-cell sample preparation

1.2.1 Preparation of *E. coli* bacteria

The procedure for the preparation of *E. coli* with the respective protein mutants was very similar to a classic expression process carried out for the purification of certain protein mutants.

Therefore, a preculture was prepared from a respective glycerol stock of BL21 DE3 bacteria. The culture was started by inoculating 20 mL Luria-Bertani (LB) supplemented with 100 µg/mL ampicillin. This culture was left shaking in an incubator overnight at 37 °C with 180 rpm. The next day 100 mL LB main cultures supplemented with 100 µg/mL ampicillin were prepared. By adding 5 mL of preculture the main cultures were inoculated. This solution was incubated at 37 °C until an OD_{600} in the range between 0.5-0.7 was reached. The cultures were then induced by the addition of 0.5 mM IPTG and the temperature was reduced to 20 °C. This solution was left for incubation overnight in the incubator. The next day cells were cooled to 3 °C on ice for 30 min and the cells were further treated in preparation for the hypotonic swelling.

1.2.2 Hypotonic swelling

The hypotonic swelling process was adapted from Hunter *et al.*[6] and the procedure was slightly modified as described in the following (Figure 1).

To harvest the cells, the cooled cultures were centrifuged for 20 min at 1600 rpm and 4 °C. The supernatant of this solution was discarded, and the cells were redissolved in 10 mL phosphate buffered saline (PBS, 8.1 mM Na_2HPO_4, 1.5 mM KH_2PO_4, 137 mM NaCl, 2.7 mM KCl, pH 7.4) buffer. Afterwards, the cells were once again centrifuged with the same parameters. The supernatant was discarded, and the cells were redissolved in another 10 mL PBS. This process was repeated once more, and the final cell pellet was resuspended in 1 mL deuterated PBS (dPBS) buffer.

The cells were then ready to be mixed in a total volume of 2 mL with 0.75 mM Cu^{II}-NTA (in dPBS buffer) and dPBS buffer. This solution was then incubated for 15 min at 37 °C in an incubator without shaking. To induce the swelling, 15 mL D_2O were added and another incubation of 15 min at 37 °C was conducted. Finally, the osmolarity of the solution was restored by adding 1.72 mL 10 × dPBS buffer and incubation for another 15 min.

To prepare an EPR sample from this solution the cells were pelleted again by centrifugation at 3200 rpm for 5 min at 4 °C. The pellet was then redissolved in 1 mL dPBS buffer, and the cells were centrifuged once more at 3200 rpm for 5 min. This process was repeated in total four times. The final cell pellet was redissolved in 85 µL dPBS buffer. 85 µL of this final solution were mixed with 56 µL d8-glycerol, resulting in a final content of 40% cryoprotectant. Of this mixture 100 µL were transferred into a 3 mm OD Quartz tube (Baumbach) via Hamilton tubing and syringe. The sample was then frozen in liquid nitrogen and stored until measurement.

1.2.3 Negative control samples

To assess potential effects of Cu^{II}-NTA that was not washed away and to exclude that something else in the studied bacterial cells contributes to the observed signals negative control samples were prepared. Therefore, to assess the background that might arise from unspecific binding of Cu^{II}-NTA to other cell components vector-less BL21 DE3 *E. coli* were used. The cells were left growing under the same conditions as described in the previous section without induction and the hypotonic swelling was carried out accordingly.

The second negative control sample was prepared in the same way as the other GB1 encoding samples, only that the hypotonic swelling was skipped by replacing the D_2O with dPBS buffer.

1.3 EPR sample preparation of *in vitro* samples and titration series

For comparison and fitting of CW data, a reference sample was made with 250 µM GB1 15H17H/28H32H, 500 µM Cu^{II}-NTA (in dPBS Buffer) and 50% d6-ethylene glycol. Of this solution 65 µL were transferred into a 3 mm OD Quartz tube (Baumbach) via Hamilton tubing and syringe. The sample was then frozen in liquid nitrogen and stored until measurement.

In order to assess the K_d of the 15H17H and 6H8H binding site, a pseudo titration series was conducted as previously described.[4] Therefore, samples with a final protein concentration of 0.5/5 μM were prepared in the presence of various amounts of Cu^{II}-NTA (Table S1). As a cryoprotectant 50% d6-ethylene glycol was added. The amount of added Cu^{II}-NTA was calculated so that the binding site occupation should be 20, 40 ,60 ,80 and 95%. The resulting solutions were transferred into a 3 mm OD Quartz tube (Baumbach) via Hamilton tubing and syringe. The samples were then frozen in liquid nitrogen and stored until measurement.

Table S1: Sample set up for the performed pseudo titration series on the GB1 mutants 15H17H/28R1 and 6H8H/28R1. The protein concentration (P) and Cu^{II}-NTA concentration (Cu^{II}-NTA) are depicted.

Mutant	c(P) / μM	c(Cu^{II}-NTA) / μM
GB1 1517H/28R1	0.5	0.2
	0.5	0.5
	0.5	0.65
	0.5	2.1
	0.5	8.5
GB1 6H8H/28R1	5	2.0
	5	4.67
	5	9.0
	5	20
	5	80.8

1.4 CW-EPR experiments

1.4.1 Instrumentation

All CW-EPR measurements were performed using a Bruker EMX 10/12 spectrometer equipped with an ELEXSYS Super Hi-Q resonator, at X-band frequencies (9.9 GHz) with a 100 kHz modulation frequency. The temperature was controlled with an ER4141 VTM Nitrogen VT unit (Bruker) operated with liquid nitrogen.

1.4.2 Low temperature measurements

The spectrometer was cooled to 120 K with liquid nitrogen. Relevant measurement parameters were adjusted for Cu^{II}-NTA respectively (Table S2).

Table S2: Measurement parameters for low temperature CW-EPR.

Variable	Value
Center field	3100 G
Sweep width	2000 G
Time constant	40.96 ms
Conversion time	20.48 ms
Modulation amplitude	4 G
Microwave power	20 mW (10 dB)
Video gain	50 dB

1.4.3 CW-Data processing and analysis

In the first instance spectra were phase and background corrected by a 9th order polynomial. Afterwards, to fit the data the measured x-axes were shifted against each other such that the zero crossings align. All spectra were aligned against an *in vitro* 250 µM GB1 15H17H/28H32H 1:2 Cu^{II}-NTA sample and normalized. This spectrum was then used as component 1 for fitting, representing the Cu^{II}-NTA bound state. The negative control sample prepared by swelling of Cu^{II}-NTA into empty *E. coli* (as described in section 1.2.3) was used as second component for fitting. Both of those were then linearly combined as described by the following equation,

$$Fit(x) = a \cdot cw_1(x) + b \cdot cw_2(x) \tag{1}$$

with *a* the fraction of component 1 (cw_1), *b* the fraction of component 2 (cw_2) and *x* the magnetic field. These parameters were constrained such that $a+b=1$ and then fitted via least squares fitting in MATLAB. Equation (1) can be rearranged the following way to calculate an error of the fit via least squares error propagation,

$$Fit = a \cdot cw_1(x) + (1-a) \cdot cw_2(x), \tag{2}$$

$$Fit = cw_2(x) + a \cdot [cw_1(x) - cw_2(x)] = cw_2(x) + a \cdot D(x) \tag{3}$$

with *D* being the difference spectrum. Additionally, residuals R_i between the target spectrum and the fit were calculated for each value of *x*,

$$R_i = cw_{Target}(x_i) - cw_{Fit}(x_i). \tag{4}$$

The variance of the noise σ_n^2 was estimated from the residual sum of squares,

$$\sigma_n^2 = \frac{1}{N-p} \sum_{i=1}^{N} R_i{}^2 \tag{5}$$

with *N* the number of data points and *p* the number of fitted parameters. These were used to calculate the variance $V(a)$ of the respective parameters and subsequently also the standard deviation $\sigma(a)$,

$$V(a) = \frac{\sigma_n^2}{\sum_i D(x_i)} \tag{6}$$

$$\sigma(a) = \sqrt{V(a)}. \tag{7}$$

The errors for both *a* and *b* are identical since the two variables are constrained.

1.5 Pulse EPR experiments

1.5.1 Instrumentation

Pulse EPR experiments were performed using a Bruker ELEXSYS 580 pulse EPR spectrometer. Temperatures were maintained using a cryogen-free variable temperature cryostat (Cryogenic Ltd) operating in the 3.5-300 K temperature range. Measurements were performed at 30 K (RIDME) and 18 K (PELDOR), using a high-power 150 W travelling-wave tube (TWT; Applied Systems Engineering) at Q-band (34 GHz) in an over coupled 3 mm cylindrical resonator (Bruker ER 5106QT-2w).

1.5.2 Relaxation time measurement

To determine the T_1 and T_m time constants, inversion recovery and 2 pulse electron spin echo decay experiments were conducted. The utilized pulse sequences are depicted in Figure S1 with the respective parameters in Table S3. To extract the resulting relaxation times mono exponential decays were fitted to the data,

$$f(T) = a \cdot \exp\left(\frac{-T}{T_1}\right) + c \tag{8}$$

$$f(2\tau) = a \cdot \exp\left(\frac{-2\tau}{T_m}\right) + c \tag{9}$$

with a and c normalization factors. The inversion recovery measurements were multiplied by -1 to allow the fitting of equation (2) to the data. In general, fitting these types of experiments with stretched exponential decays in case of the 2p echo decays and biexponential decays for the inversion recovery has been shown to give more accurate values for the respective relaxation times.

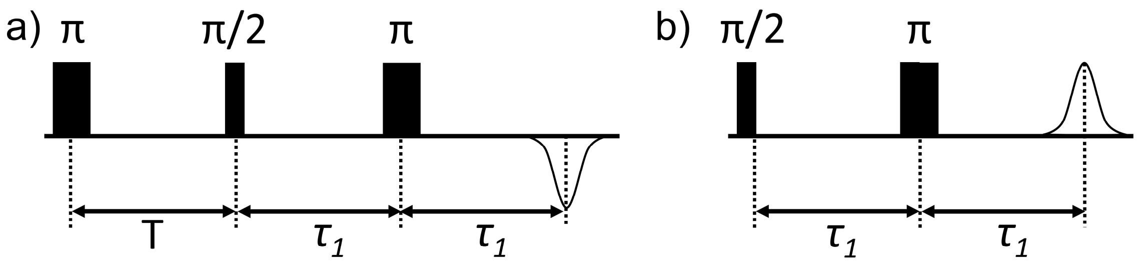

Figure S1: Pulse sequences of the a) Inversion recovery experiment, and b) 2p echo decay experiment.

Table S3: Parameters of the 2p echo decay and inversion recovery experiment.

Variable	Inversion recovery	2p echo decay
(π/2)	12 ns	8 ns
(π)	24 ns	16 ns
$(\pi)_{inv.}$	24 ns	-
τ_1	400 ns	400 ns
T_{start}	1000 ns	-
$T_{increment}$ / $\tau_{2,\ increment}$	500 ns	16 ns
Shot Repetition Time (SRT)	1 ms	400 µs

1.5.3 PELDOR measurement

Four pulse pulsed electron-electron double resonance (PELDOR)[7,8] experiments were performed using the pulse sequence depicted below (Figure S2) using a 16-step phase cycle and a 16/17-step τ_1/τ_2 modulation averaging with 8 ns length, depending on the field position. Measurements were conducted at 18 K using the parameters listed below (Table S4). The detection frequency was applied with a frequency offset of -150 MHz with respect to the pump frequency. The pulses were set up via the AWG, for detection rectangular pulses and as the pump pulse a HS {1,1} pulse were used. To remove the effects of orientation selection, measurements were conducted at field positions -100 G, -540 G and -830 G from the maximum field positions.[9] The individual traces were summed up and weighted by the respective echo detected field sweep (EDFS) intensity.

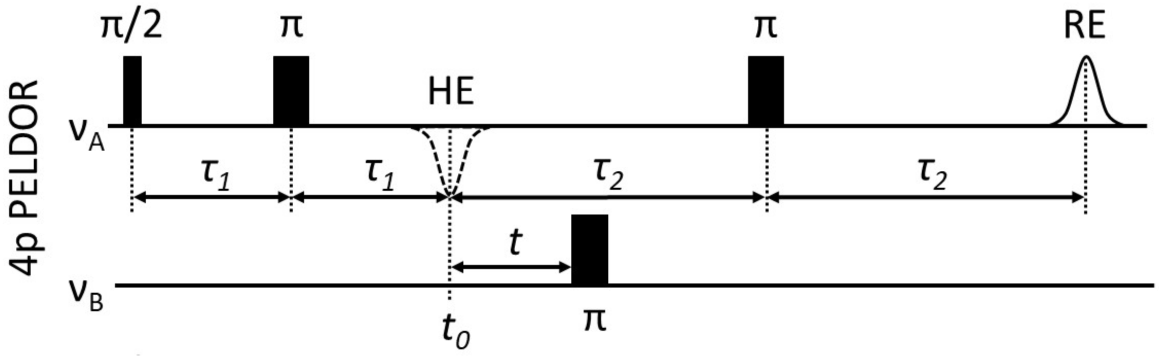

Figure S2: 4 pulse PELDOR sequence, with the Hahn echo (HE) and the refocussed echo (RE).

Table S4: Parameters of the 4 pulse PELDOR experiment.

Variable	Value
$(\pi/2)_A$	16 ns
$(\pi)_A$	32 ns
τ_1	300 ns
τ_2	1900 ns
Shot Repetition Time (SRT)	1 ms
$(\pi)_B$ length	150 ns
$(\pi)_B$ width	150 MHz
$(\pi)_B$ offset	150 MHz

1.5.4 RIDME measurement

The utilized parameters of the relaxation induced dipolar modulation enhancement (RIDME)[10,11] experiment (Figure S3) are depicted in Table S5. T_{mix} and shot repetition times at 30 K were adapted from a previous publication.[1] A 8-step phase cycle and a 16/17-step τ_1/τ_2 modulation averaging with 8 ns length was applied, for low fields 17-steps and for higher fields around the maximum of the Cu^{II}-spectrum 16-steps were utilized. All RIDME traces were recorded with a short (5 μs) and a long (35 μs) mixing time for deconvolution. To remove the effects of orientation selection, measurements were conducted at field

positions -100 G, -540 G and -830 G from the maximum field position.[12] The individual traces were summed after deconvolution and weighted by the respective EDFS intensity.

When RIDME measurements were conducted on R1 Cu^{II}-NTA mixed mutants the measurement was set up on the maximum of the nitroxide EDFS. An 8-step phase cycle and 8-step τ_1/τ_2 modulation averaging with 16 ns length was applied. Further parameters for this set up are depicted in Table S5.

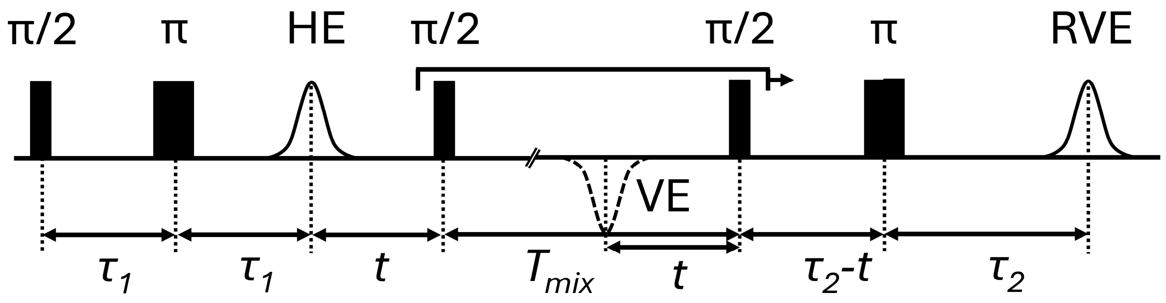

Figure S3: 5 pulse RIDME sequence, with the Hahn echo (HE), the virtual echo (VE) and the refocused virtual echo (RVE) which is used for detection.

Table S5: Parameters of the 5 pulse RIDME experiment.

Variable	Cu-Cu	Cu-R1
π/2	8 ns	8 ns
π	16 ns	16 ns
T_{mix}	5 / 35 µs	5 / 200 µs
t	-100 ns	-100 ns
τ_1	400 ns	400 ns
τ_2	1900 ns	2000 ns
Shots per point (SPP)	3	1
Shot Repetition Time (SRT)	400 µs	30 ms

1.5.5 PDS data processing and analysis

Four pulse PELDOR and RIDME data was analyzed using the ComparitiveDEERAnalyzer2.0 (CDA2.0) in DeerAnalysis2022.[13,14] If necessary, up to 5% of the trace were cut off at the end in order to remove artifacts. For some RIDME and PELDOR traces even larger cut-offs of about 50% were necessary to allow analysis with the CDA2.0, due to the more complex background shape of some of the measured in-cell traces. In all cases where CDA data is presented the consensus distribution and fit traces are shown. To assess the signal to noise ratio (SNR), the modulation depth Δ of PELDOR and RIDME traces was adapted from the CDA2.0 output. The noise was calculated as root mean square deviation (RMSD), estimated from the second and third quartile of the imaginary part of the phase-corrected trace.[1] The SNR was then weighted by the square root of the acquisition time *t*,

$$SNR_t = \frac{\Delta}{RMSD} \cdot \frac{1}{\sqrt{t}}. \qquad (10)$$

1.6 Mass spectrometry

ESI mass spectrometry was performed on samples before and after labeling. Samples were diluted to 1 µM in 1% formic acid (FA). 30 pmol per sample were injected onto the liquid chromatography (LC) system with a time of flight (TOF) mass spectrometer (Xevo G2 TOF MS with Acquity HPLC, Waters) using a MassPrep cartridge column (Waters), applying a 5 min gradient from 95% water, 5% acetonitrile to 5% water, 95% acetonitrile (eluents supplemented with 1% FA). Data was collected in positive mode from 500-2500 m/z, and charged ion series deconvolution to 0.1 Da resolution was performed using the MaxEnt I algorithm utilizing a peak width at half height of 0.5 m/z.

2. Results and discussion

2.1 Protein purification

The last purification step via size exclusion chromatography (SEC) of the GB1 (Figure S4) mutants are shown, confirming their purity. The identity was controlled via mass spectrometry in section 2.2 (15H17H/28C) and Figure S5 (15H17H/28H32H).

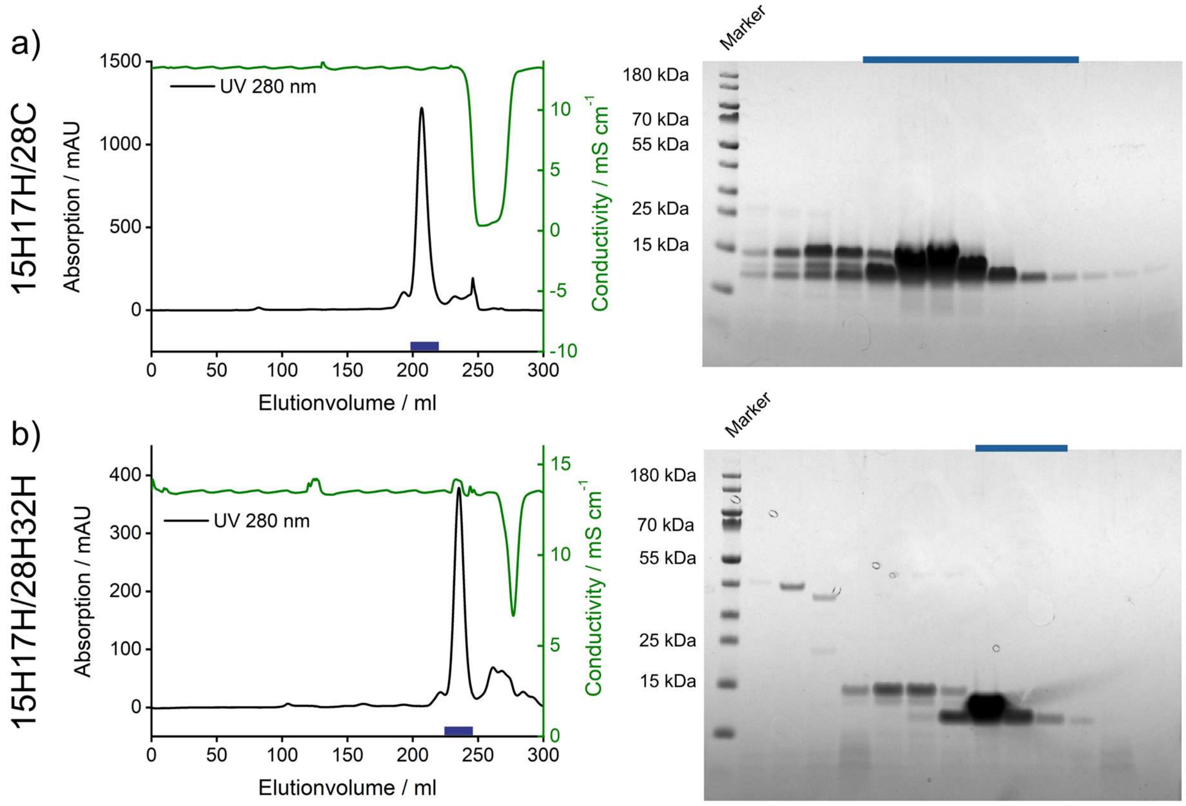

Figure S4: SEC chromatogram of the last purification step (left) and 4-12% NuPAGE gel image (right). The pooled fractions and their respective positions in the chromatogram are marked in blue. GB1 mutants, a)15H17H/28C and b) 15H17H/28H32H.

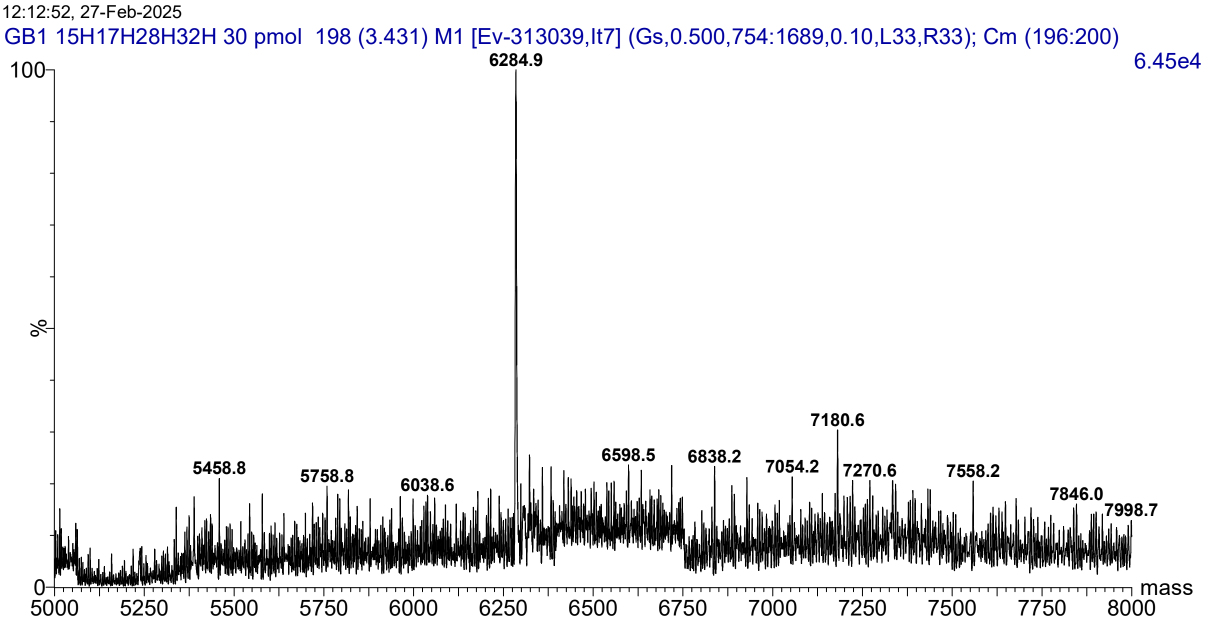

Figure S5: Deconvoluted ESI mass spectrometry data for GB1 15H17H/28H32H. The measured mass of 6284.9 Da agrees with the calculated mass of 6284.88 Da.

2.2 MTSL labeling

The successful labeling of the cysteine mutants was confirmed by ESI mass spectrometry (Figure S6+S7). The measured masses agree within ±2 Da with the calculated masses as depicted in Table S6. Only the mass for GB1 6H8H/28R1 mutants varies by a larger margin, because this measurement was conducted in deuterated solution. This leads to partial exchange of protons to deuterons affecting the accuracy of the exact mass.

Table S6: Calculated and measured masses for all samples with and without MTSL.

Mutant	Calculated mass / Da	Measured mass / Da
GB1 15H17H/28C	6241.87	6241.8
GB1 15H17H/28R1	6427.17	6424.9
GB1 6H8H/28C	6244.82	6245.0
GB1 6H8H/28R1	6430.12	6444.2

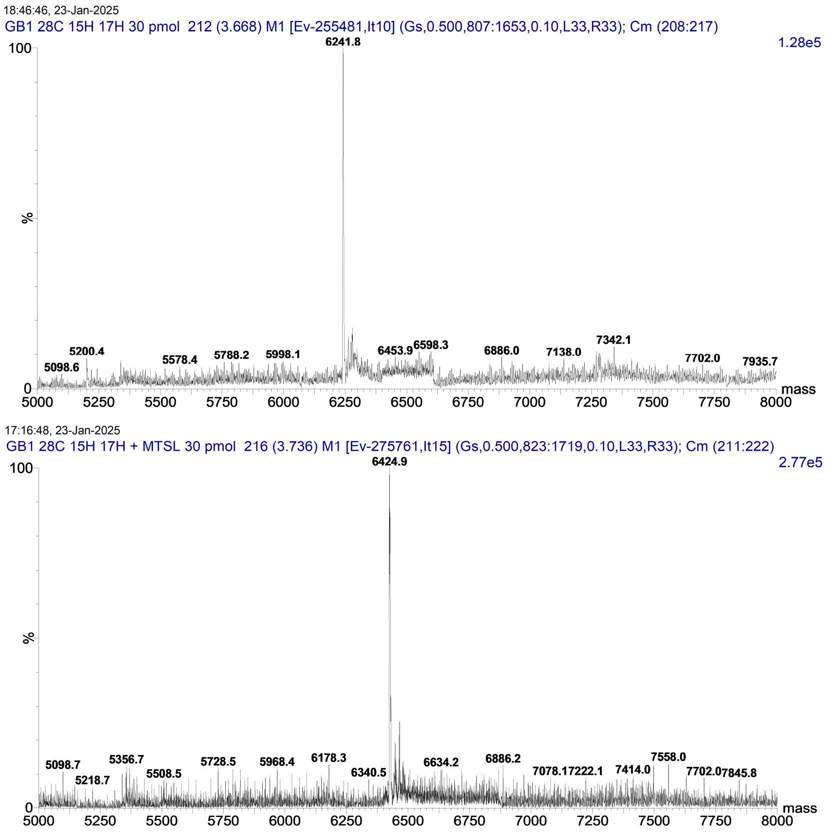

Figure S6: Deconvoluted ESI mass spectrometry data for GB1 15H17H/28C (top) and 15H17H/28R1 (bottom).

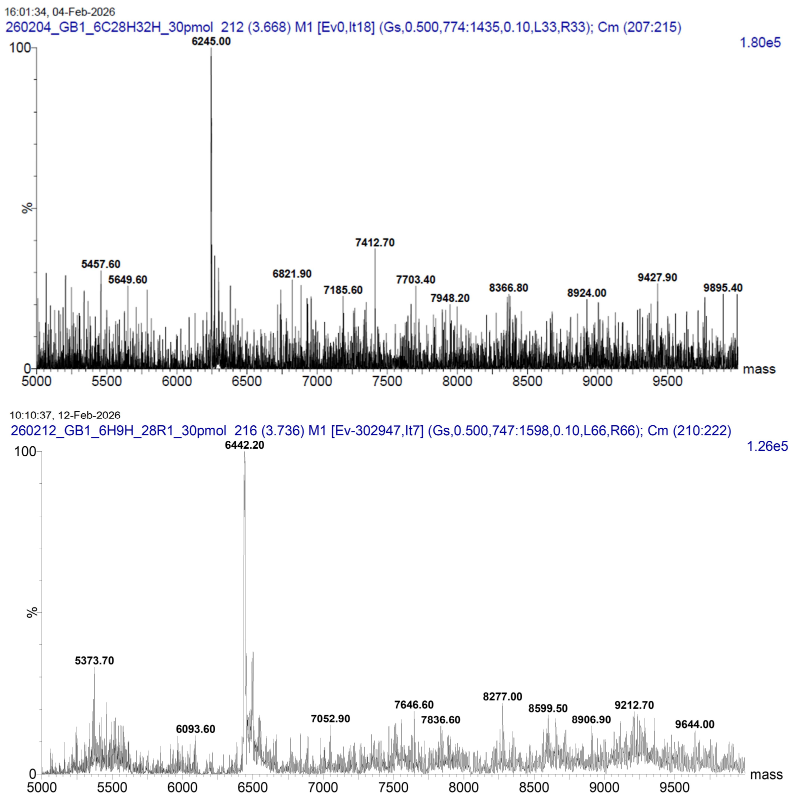

Figure S7: Deconvoluted ESI mass spectrometry data for GB1 6H8H/28C (top) and 6H8H/28R1 (bottom).

2.3 Pseudo titration series, additional information

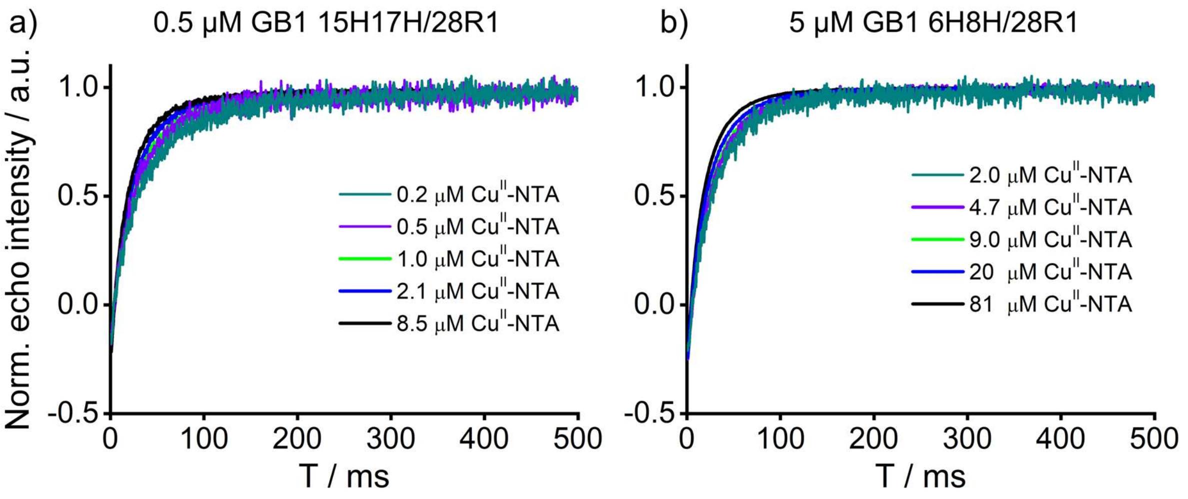

Figure S8: Inversion recovery measurement of samples in Figure 2, measured at 30 K. a) 0.5 µM GB1 15H17H/28R1 and b) 5 µM GB1 6H8H/28R1.

The mono-exponentially fitted T_1 values for the inversion recovery measurements are shown in Table S7. Additionally, the modulation depth (Δ) fits from the CDA2.0 output are depicted in the same Table.

Table S7: Fitted modulation depths (Δ) from the deconvoluted RIDME traces and mono exponential T_1 fits to the data in Figure S8. The standard deviation (σ) from the fits is depicted in case of the T_1 fits.

Mutant	c(Cu^{II}-NTA) / µM	Δ (decon.)	T_1 / µs
GB1 15H17H/28R1	0.2	0.113	42.0 ± 0.5
	0.5	0.296	32.6 ± 0.4
	0.95	0.375	30.3 ± 0.2
	2.1	0.408	26.1 ± 0.1
	8.5	0.420	22.5 ± 0.1
GB1 6H8H/28R1	2	0.120	31.4 ± 0.3
	4.67	0.251	28.7 ± 0.2
	9	0.365	27.0 ± 0.1
	20	0.425	24.2 ± 0.1
	80.8	0.451	20.8 ± 0.1

Based on the utilized T_{mix} and fitted T_1 values, the maximal modulation depth (Δ_{Tmix}) was calculated as described earlier.[4] The percentage loading used for the isotherm fit in the main text (Figure 2) was calculated from the ratio Δ/Δ_{Tmix}. The K_d was then fitted to the data with the previously described[4] quadratic binding equation:

$$\Delta\Delta_{Tmix}^{-1} = \frac{(K_D+[P]_t+[M]_t)-\sqrt{(K_D+[P]_t+[M]_t)^2-4[P]_t[M]_t}}{2[P]_t} \qquad (11)$$

with $[P]_t$ the total protein concentration and $[M]_t$ the total metal complex concentration. The resulting K_d values are depicted in Figure 2 in the main text. The error of the K_d values

was estimated from the uncertainties in the T_1 fits and assuming an uncertainty of 0.01 in the modulation depth determination. The CDA2.0 fits of the RIDME traces in main text Figure 2 are depicted below in Figure S9.

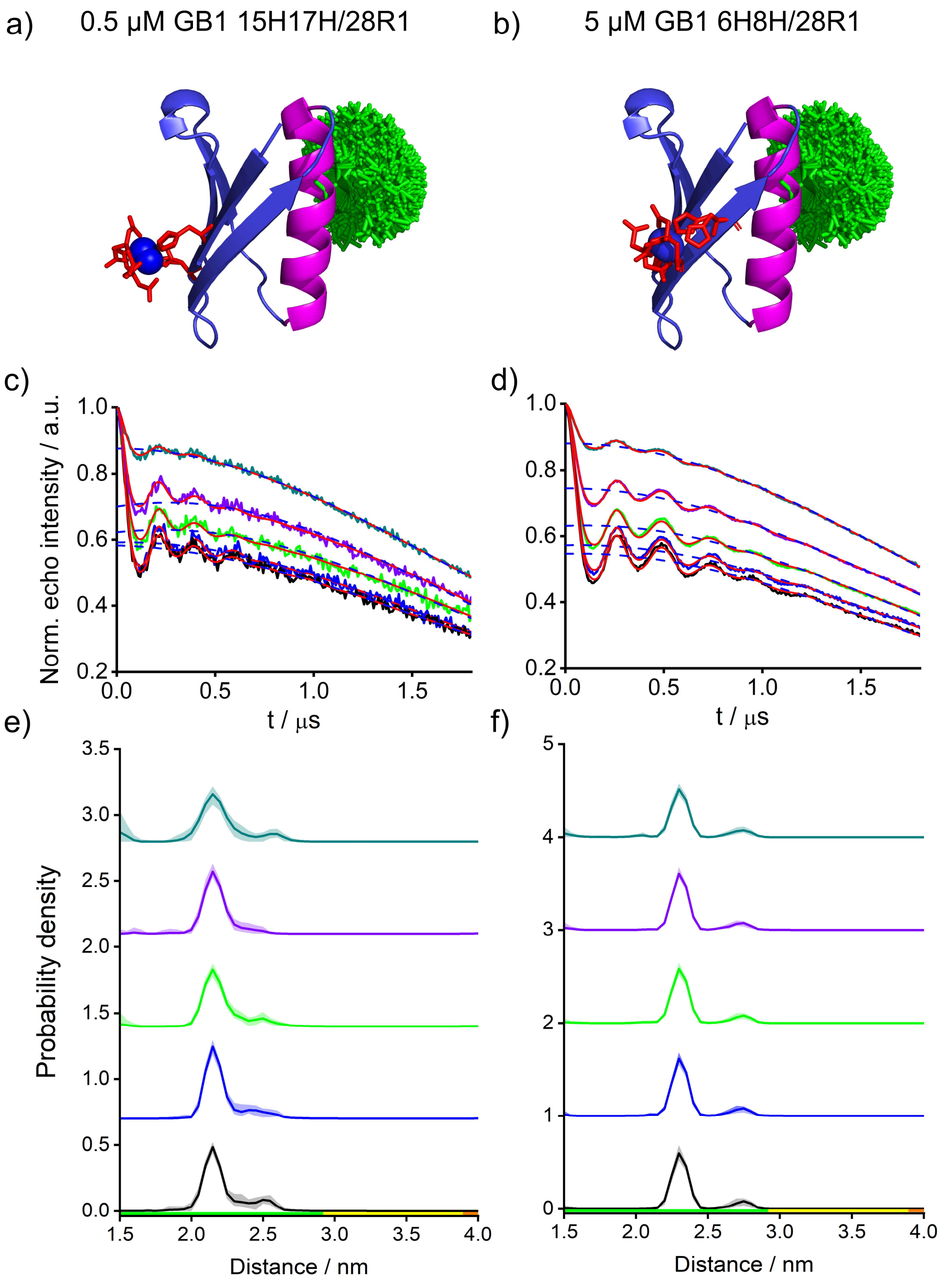

Figure S9: Fitted traces and distance distributions of the data presented in Figure 2 main text. a) A crystal structure of GB1 (PDB: 4WH4) with the rotamer cloud of the MTSL labelled 28C mutant and the Cu^{II}-NTA labelled 15H17H dHis motif, generated in ChiLife.[15,16] b) As in a) for the GB1 6H8H/28R1 mutant. c) Deconvoluted RIDME measurements on the maximum of the MTSL echo detected field sweep spectrum (EDFS) of 0.5 µM GB1 15H17H/28R1 with increasing Cu^{II}-NTA concentrations and the fits extracted from the CDA2.0 analysis. d) Deconvoluted RIDME measurements on the maximum of the MTSL EDFS spectrum of 5 µM GB1 6H8H/28R1 with increasing Cu^{II}-NTA concentrations and the fits extracted from the CDA2.0 analysis. e+f) Consensus distance distributions resulting from the fits in (c) and (d) respectively as well as a color bar from green to orange depicting the reliability of the distance distribution.

2.4 Negative control samples

To assess whether there is any unspecific binding of Cu^{II}-NTA to anything else than GB1, a hypotonic swelling experiment with bacteria not bearing the GB1 gene containing plasmid was conducted. The resulting expression gel (Figure S10a) shows that there was no GB1 expression. When looking at the CW-EPR spectrum (Figure S10b, green spectrum) some copper signal can be seen, which is much smaller in intensity than the signal of the sample that expressed GB1. The resulting 2p echo decay measurement (Figure S10c) is comparably similar to the reference, whereas the resulting RIDME trace (Figure S10d) features only a fast decay without any visible dipolar oscillation. This suggests that the background resulting from swelling of Cu^{II}-NTA in the absence of high affinity binding sites is negligible.

To exclude potential leakage of protein out of the cells *E. coli* that did express GB1 were incubated with Cu^{II}-NTA without inducing the hypotonic swelling (Figure S10a). The sample does not show a significant Cu^{II} signal in the CW spectra (Figure S10b, blue spectrum), only features that can be attributed to manganese are visible. This is confirmed when measuring 2p echo decays and RIDME measurements at the expected Cu^{II} field position. The echo decay is slower and the signal in general is much weaker than for the reference sample (Figure S10c, blue trace). Furthermore, the RIDME (Figure S10d, blue trace) does not show any dipolar oscillation or modulation depth. This confirms that there is no residual Cu^{II}-NTA that has bound to the outer surface of the bacteria or to protein that leaked out of the bacteria.

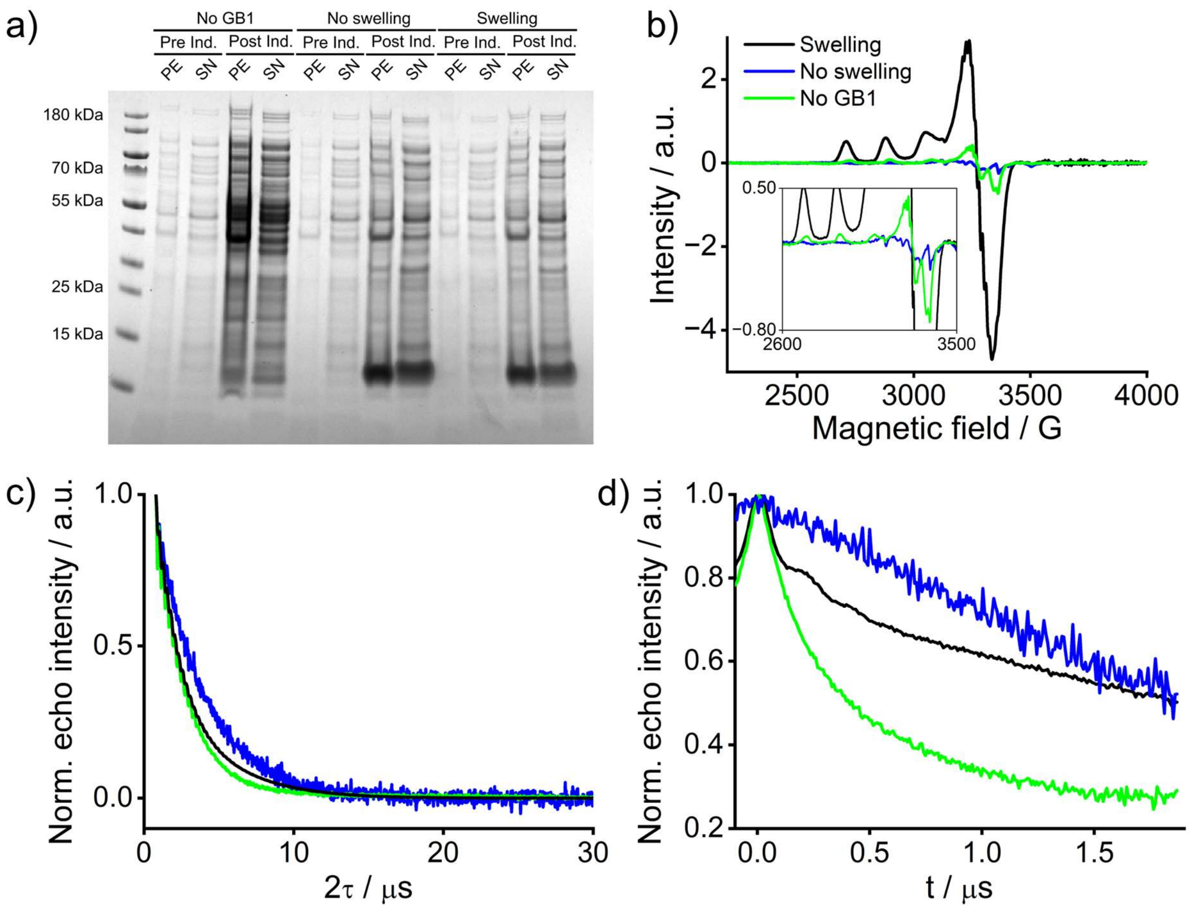

Figure S10: Negative control samples prepared without swelling (blue), with *E. coli* bacteria that did not express GB1 15H17H/28H32H (green) and a reference sample that was prepared with swelling and GB1 15H17H/28H32H expression (black). a) 4-12% NuPAGE gel of samples before (Pre Ind.) and after (Post Ind.) induction of protein expression. The insoluble fraction (PE) and soluble fraction (SN) are shown. b) CW-EPR measurement at 120 K. c) 2p echo decay measurements as well as d) RIDME measurements at 30 K.

2.5 Additional tests

2.5.1 Influence of prolonged incubation times

To test whether signal decay or reduction of the Cu^{II}-NTA is taking place, another set of samples was prepared by increasing the final incubation step in dPBS Buffer (Figure 1) from 15 min up to 4 h. For each time point an individual sample was prepared and the cell viability assessed (Figure S11b). Therefore, serial dilutions of 1:40 were made from the respective solutions. These were compared against bacteria that were not subjected to hypotonic swelling. The viability is only marginally affected by the prolonged incubation period. Also, the Cu^{II}-NTA and the use of deuterated solutions does not affect the cell viability (Figure S11b, row 1 and 2). The signal originating from the Cu^{II}-NTA seems to decrease slowly over time (c). Even a very stable Cu^{II}-NTA complex will have an off rate leading to a slow disassembly, which might explain the observed signal decay. This process might be faster when the cells are incubated in growth medium instead of dPBS buffer, which remains to be tested. It was possible to perform RIDME measurements

(Figure S11e) on all samples. First and foremost a decrease in modulation depth was observed and after 30 min of incubation an increasingly steep background decay.

These results seem to highlight the importance of very tight binding of the Cu^{II}-NTA complex to slow down the dissociation of the complexes and following reduction processes. In complexes with a higher dissociation constant these processes might happen on a faster time scale, effectively hindering successful distance measurements.

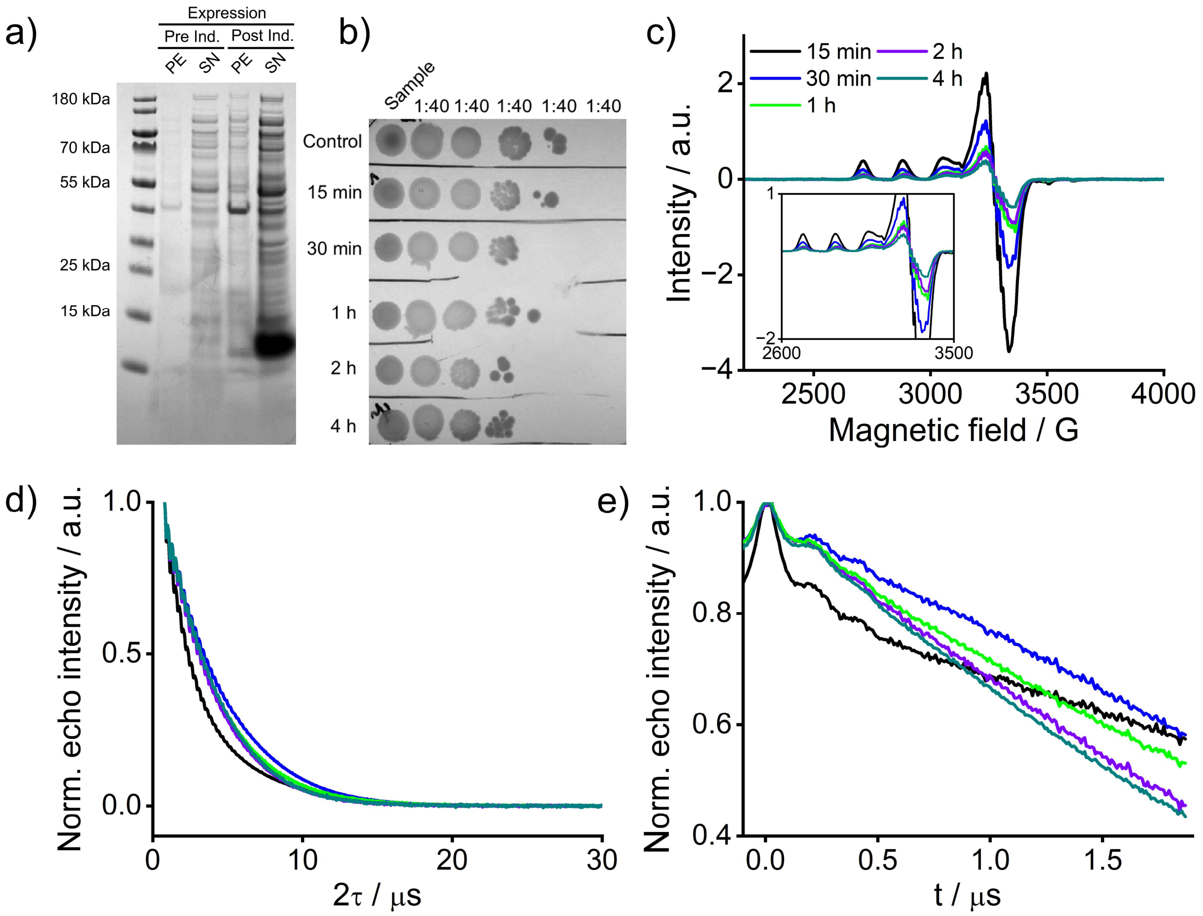

Figure S11: Time dependent samples of overexpressed GB1 15H17H/28H32H in *E. coli* prepared after 15 min (black), 30 min (blue), 1 h (green), 2 h (violet) and 4 h (dark cyan) incubation in dPBS. a) 4-12% NuPAGE gel of samples before (Pre Ind.) and after (Post Ind.) induction of protein expression. The insoluble fraction (PE) and soluble fraction (SN) are shown. b) Cell viability assay, showing a control (bacteria that were only washed in PBS Buffer) and the respective samples after different incubation times. The initial solution was diluted 1:40 five times. b) CW-EPR measurement at 120 K. c) 2p echo decay measurements as well as d) RIDME measurements at 30 K.

2.5.2 Expression time experiment

To assess the effects of the protein expression levels on the experiment a set of samples was prepared with increasing expression time before the hypotonic swelling procedure. As the expression gel (Figure S12a) clearly shows, the amount of GB1 (slightly below 15 kDa marker) increases gradually and is easily visible at about 1 h of expression. After 4 h a significant amount was already expressed. Nevertheless, the resulting CW measurements (Figure S12b) did not show the expected gradual increase in signal

intensity. The signal does increase from 15 min expression to 4 h but is still substantially smaller than the signal of the sample that was expressed overnight. Also, the 4 h sample does not show any dipolar oscillation in the RIDME measurement (Figure S12b). This result seems to point towards the necessity to have a large excess of expressed material and thereby also dHis sites. A second possible explanation would be, that the bacteria are in a less active state due to depletion of the nutrients in the growth medium. These effects cannot be disentangled from the data at hand and require further investigations.

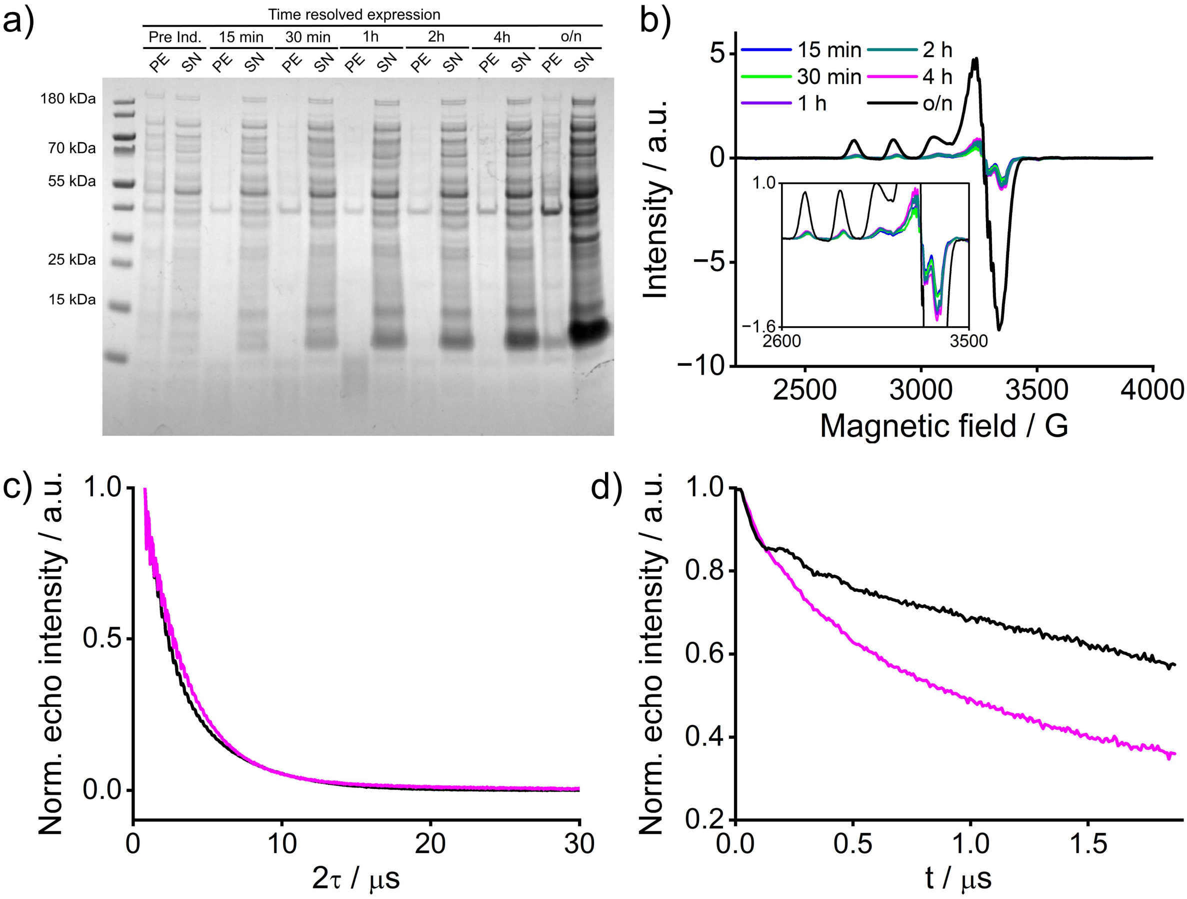

Figure S12: Time dependent samples prepared after 15 min (blue), 30 min (green), 1 h (violet), 2 h (dark cyan), 4 h (magenta) and o/n (black) expression of GB1 15H17H/28H32H in *E. coli*. a) 4-12% NuPAGE gel of samples before (Pre Ind.) and after (Post Ind.) induction of protein expression. The insoluble fraction (PE) and soluble fraction (SN) are shown. b) CW-EPR measurement at 120 K. c) 2p echo decay measurements as well as d) RIDME measurements at 30 K.

2.5.3 CW fits of double dHis mutants and repeats

The fitting procedure for CW spectra described in section 1.4.3 and utilized in main text Figure 3 has been employed to fit the spectra of in-cell samples of GB1 15H17H/28H32H (Figure S13) and GB1 6H8H/28H32H (Figure S14). Additionally, repeats of both samples were fitted as well.

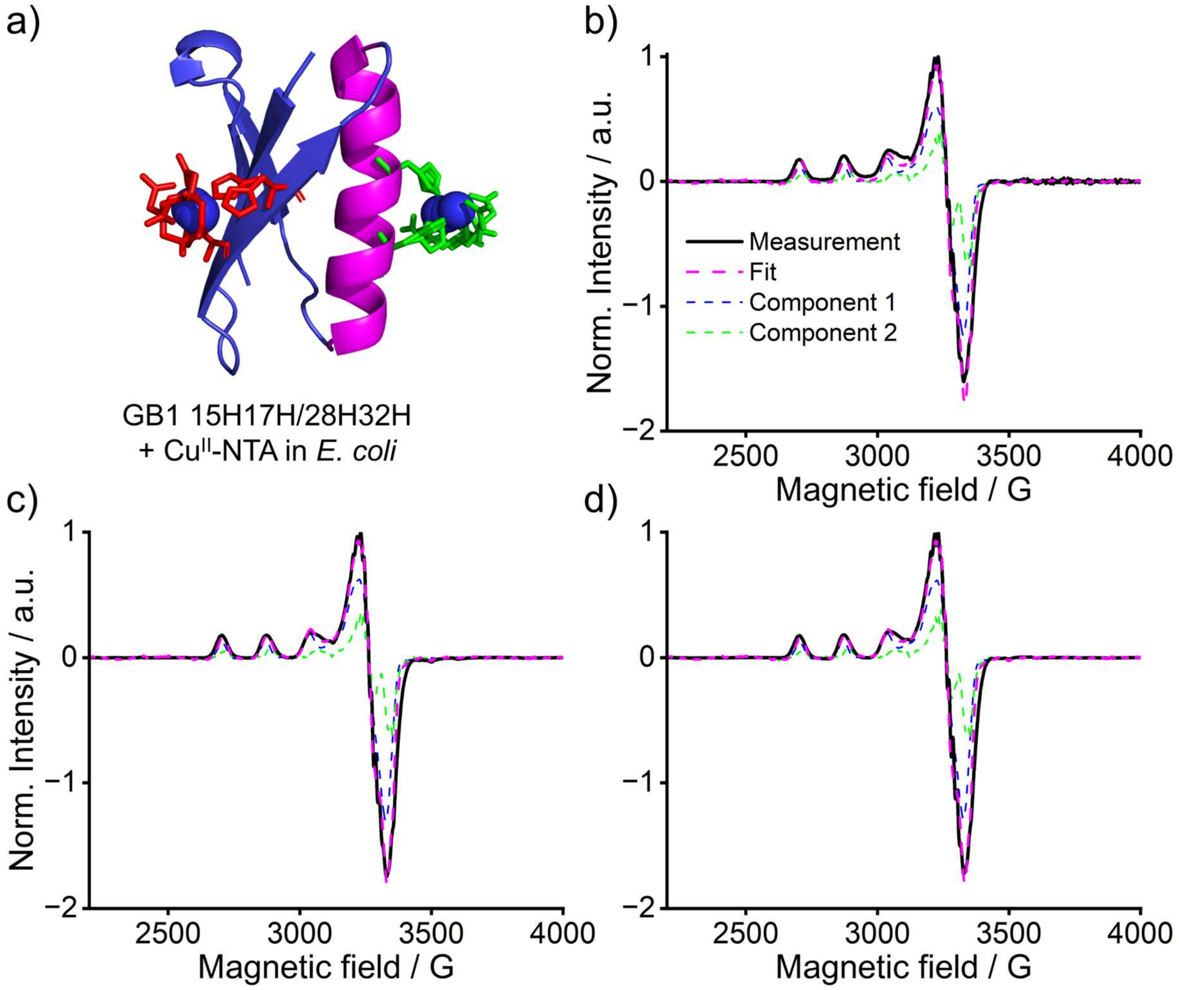

Figure S13: Investigation of the GB1 15H17H/28H32H *E. coli* sample swelled with Cu^{II}-NTA in Figure 4 and biological repeats, via CW-EPR spectroscopy measured at 120 K. a) The dHis sites labelled with Cu^{II}-NTA in ChiLife. b) The sample utilized in Figure 4. Depicted is the measurement (black), the fit (magenta, dash), the fraction of component 1 (blue, dash) and the fraction of component 2 (green, dash). c) First biological repeat of the same mutant in the color code in (b). d) Second biological repeat of the same mutant in the color code in (b).

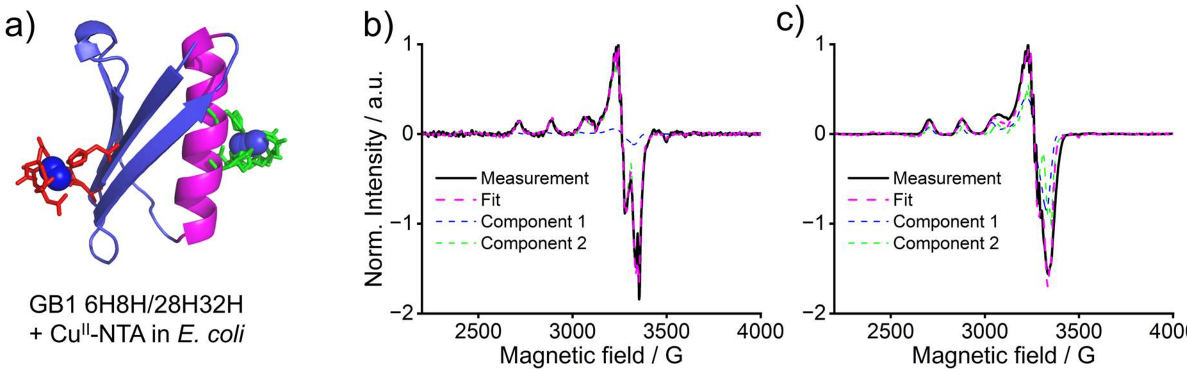

Figure S14: Investigation of the GB1 6H6H/28H32H *E. coli* sample swelled with Cu^{II}-NTA in Figure 1 and a biological repeat, via CW-EPR spectroscopy measured at 120 K. a) The dHis sites labelled with Cu^{II}-NTA in ChiLife. b) The sample utilized in Figure 1. Depicted is the measurement (black), the fit (magenta, dash), the fraction of component 1 (blue, dash) and the fraction of component 2 (green, dash). c) First biological repeat of the same mutant in the color code in (b).

The resulting fractions of the respective components are summarized in Table S8. Looking at the trend for the double dHis mutants the respective fraction of component 1 lies in between the respective single dHis mutant, i.e. the 15H17H/28H32H samples feature values of about 0.6 whereas the 28H32H alone can be fitted with 0.42 component 1 and the 15H17H with 0.911. The same is true for the GB1 6H8H/28H32H mutant, the only difference is the strongly varying fraction of component 1 between the samples. This might be explained by the lower affinity of the binding sites in this mutant. The resulting uncertainties appear low. However, it needs to be noted that the variability between the repeats is significantly higher, suggesting a higher uncertainty in the sample preparation procedure than in the fitting.

Table S8: Values for the respective fractions a (component 1) and b (component 2) of the fits to the data in Figure 3 and Figure S13+14. The standard deviations (σ) of the fits are depicted for the parameters.

Mutant	a	b
GB1 6H8H	0.262 ± 0.008	0.738 ± 0.008
GB1 28H32H	0.420 ± 0.006	0.580 ± 0.006
GB1 15H17H/28C	0.911 ± 0.002	0.089 ± 0.002
GB1 6H8H/28H32H	0.057 ± 0.003	0.943 ± 0.003
GB1 6H8H/28H32H 1st repeat	0.406 ± 0.005	0.594 ± 0.005
GB1 15H17H/28H32H	0.593 ± 0.005	0.407 ± 0.005
GB1 15H17H/28H32H 1st repeat	0.625 ± 0.004	0.375 ± 0.004
GB1 15H17H/28H32H 2nd repeat	0.617 ± 0.005	0.383 ± 0.005

2.6 PELDOR and RIDME comparison

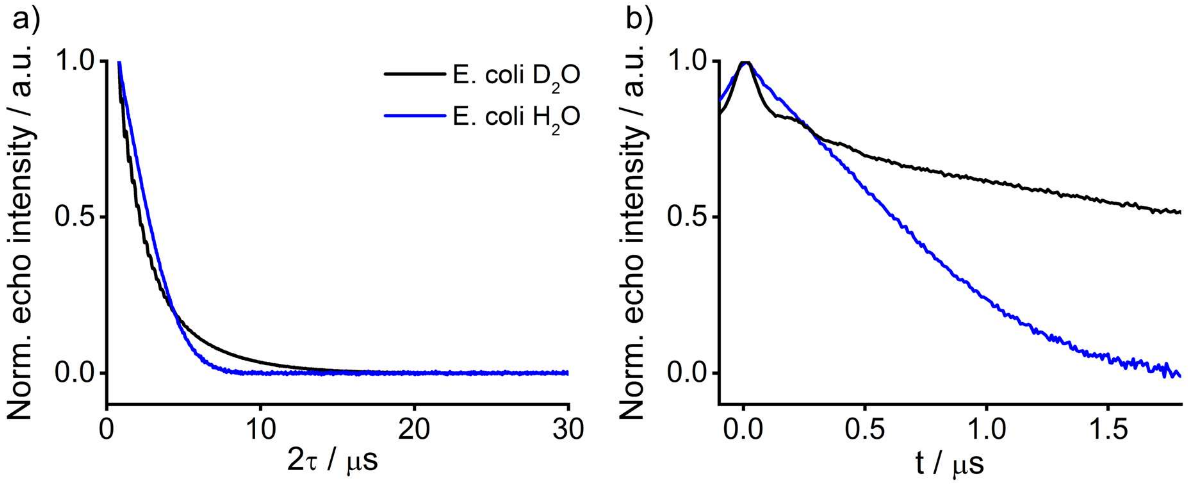

Figure S15: Comparison of swelling with D_2O and H_2O of the overexpressed GB1 15H17H/28H32H mutant, measured at 30 K. a) 2p echo decay measurements and b) Summed RIDME measurements.

Even though the initial decay of the sample that has been swelled in D_2O (Figure S15a, black trace) is faster than the H_2O equivalent, at longer τ values the signal decays significantly slower. The fitted relaxation times (Table S9) confirm this trend, in favor of the deuterated sample. Interestingly when comparing the RIDME experiments (Figure S15b), the background decays much quicker in the H_2O sample and the oscillation in the trace is not visible. This suggests that RIDME measurements in this environment need some degree of deuteration to produce analyzable results.

Table S9: T_m relaxation times, fitted with mono exponential fits of the 2p echo decay measurements in Figure S15a. The standard deviation (σ) from the fit is depicted in case of the T_m fits.

**Sample**	**T_m / μs**
D_2O	2.33 ± 0.01
H_2O	2.16 ± 0.01

2.7 Biological repeats RIDME measurements on GB1 15H17H/28H32H

The biological repeats depicted in Figure S16 demonstrate that the conducted experiment yields comparable results. The average distances vary by less than 1 Å and the shape of the resulting distance distributions is comparable. Some deviations are visible, such as variations in modulation depth as well as differences in the speed of background decay. This is especially pronounced for repeat 2 which also depicts a higher standard deviation of the average distances. However, the main distance peak at about 2.2 nm is present in all samples.

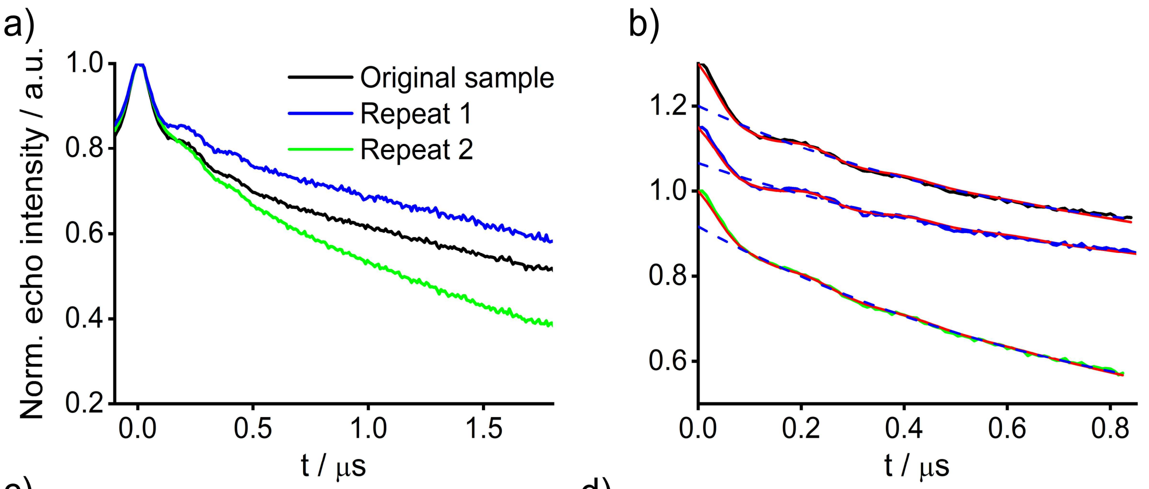

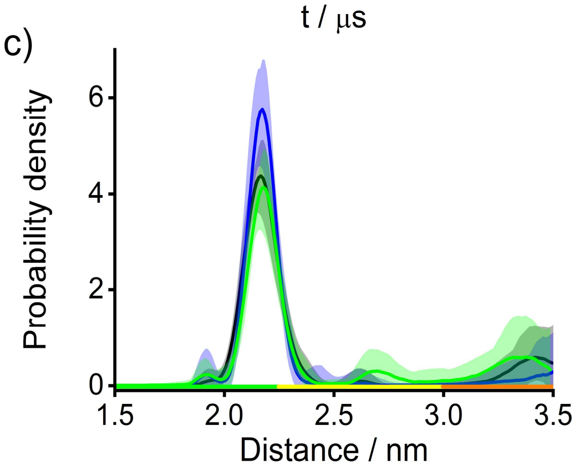

d)

Sample	Δ	<r> / nm	σ_r / nm
Original	0.103	2.18	0.1
Repeat 1	0.087	2.17	0.06
Repeat 2	0.087	2.14	0.91

Figure S16: Biological repeats of the GB1 15H17H/28H32H mutant in *E. coli* swelled with D_2O. a) Summed and deconvoluted RIDME traces for the sample depicted in Figure 4 main text (black), the 15 min sample depicted in Figure S11 (Repeat 1, blue) and a second repeat (Repeat 2, green). b) The fits (red) and backgrounds (blue dashed) extracted from the CDA2.0 analysis, in the same color code as in (a). c) Calculated distance distributions resulting from the fits in (b), in the same color code as in (a). A color bar from green to orange depicting the reliability of the distance distribution is shown. d) Deconvoluted modulation depth (Δ), average distances (<r>) and standard deviations of the average distance (σ_r) extracted from the CDA2.0 report.

3. Literature

(1) Ackermann, K.; Wort, J. L.; Bode, B. E. Nanomolar Pulse Dipolar EPR Spectroscopy in Proteins: Cu^{II}-Cu^{II} and Nitroxide-Nitroxide Cases. *J. Phys. Chem. B.* **2021**, *125* (20), 5358–5364.
(2) Cunningham, T. F.; Putterman, M. R.; Desai, A.; Horne, W. S.; Saxena, S. The double-histidine Cu^{2+}-binding motif: a highly rigid, site-specific spin probe for electron spin resonance distance measurements. *Angew. Chem., Int. Ed.* **2015**, *54* (21), 6330–6334.
(3) Ghosh, S.; Lawless, M. J.; Rule, G. S.; Saxena, S. The Cu^{2+}-nitrilotriacetic acid complex improves loading of α-helical double histidine site for precise distance measurements by pulsed ESR. *J. Magn. Reson.* **2018**, *286*, 163–171.
(4) Wort, J. L.; Ackermann, K.; Giannoulis, A.; Stewart, A. J.; Norman, D. G.; Bode, B. E. Sub-Micromolar Pulse Dipolar EPR Spectroscopy Reveals Increasing Cu^{II} -labelling of Double-Histidine Motifs with Lower Temperature. *Angew. Chem., Int. Ed.* **2019**, *58* (34), 11681–11685.
(5) Schiemann, O.; Heubach, C. A.; Abdullin, D.; Ackermann, K.; Azarkh, M.; Bagryanskaya, E. G.; Drescher, M.; Endeward, B.; Freed, J. H.; Galazzo, L.; Goldfarb, D.; Hett, T.; Esteban Hofer, L.; Fábregas Ibáñez, L.; Hustedt, E. J.; Kucher, S.; Kuprov, I.; Lovett, J. E.; Meyer, A.; Ruthstein, S.; Saxena, S.; Stoll, S.; Timmel, C. R.; Di Valentin, M.; Mchaourab, H. S.; Prisner, T. F.; Bode, B. E.; Bordignon, E.; Bennati, M.; Jeschke, G. Benchmark Test and Guidelines for DEER/PELDOR Experiments on Nitroxide-Labeled Biomolecules. *J. Am. Chem. Soc.* **2021**, *143* (43), 17875–17890.
(6) Hunter, H. R.; Kankati, S.; Hasanbasri, Z.; Saxena, S. Endogenous Cu(II) Labeling for Distance Measurements on Proteins by EPR. *Chem. Eur. J.* **2024**, *30* (72), e202403160.
(7) Martin, R. E.; Pannier, M.; Diederich, F.; Gramlich, V.; Hubrich, M.; Spiess, H. W. Determination of End-to-End Distances in a Series of TEMPO Diradicals of up to 2.8 nm Length with a New Four-Pulse Double Electron Electron Resonance Experiment. *Angew. Chem., Int. Ed.* **1998**, *37* (20), 2833–2837.
(8) Milov, A. D.; Salihkov, K. M.; Shirov, M. D. Application of ELDOR in electron-spin echo for paramagnetic center space distribution in solids. *Fiz. Tverd. Tela* **1981**, *23* (4), 975–982.
(9) Bogetti, X.; Hasanbasri, Z.; Hunter, H. R.; Saxena, S. An optimal acquisition scheme for Q-band EPR distance measurements using Cu^{2+}-based protein labels. *Phys. Chem. Chem. Phys.* **2022**, *24* (24), 14727–14739.
(10) Milikisyants, S.; Scarpelli, F.; Finiguerra, M. G.; Ubbink, M.; Huber, M. A pulsed EPR method to determine distances between paramagnetic centers with strong spectral anisotropy and radicals: the dead-time free RIDME sequence. *J. Magn. Reson.* **2009**, *201* (1), 48–56.
(11) Kulik, L. V.; Dzuba, S. A.; Grigoryev, I. A.; Tsvetkov, Y. Electron dipole–dipole interaction in ESEEM of nitroxide biradicals. *Chem. Phys. Lett.* **2001**, *343* (3-4), 315–324.

(12) Heubach, C. A.; Hasanbasri, Z.; Abdullin, D.; Reuter, A.; Korzekwa, B.; Saxena, S.; Schiemann, O. Differentiating between Label and Protein Conformers in Pulsed Dipolar EPR Spectroscopy with the dHis-Cu^{2+} (NTA) Motif. *Chem. Eur. J.* **2023**, *29* (72), e202302541.
(13) Jeschke, G.; Chechik, V.; Ionita, P.; Godt, A.; Zimmermann, H.; Banham, J.; Timmel, C. R.; Hilger, D.; Jung, H. DeerAnalysis2006—a comprehensive software package for analyzing pulsed ELDOR data. *Appl. Magn. Reson.* **2006**, *30* (3-4), 473–498.
(14) Worswick, S. G.; Spencer, J. A.; Jeschke, G.; Kuprov, I. Deep neural network processing of DEER data. *Sci. Adv.* **2018**, *4* (8), eaat5218.
(15) Tessmer, M. H.; Stoll, S. chiLife: An open-source Python package for in silico spin labeling and integrative protein modeling. *PLoS Comput. Biol.* **2023**, *19* (3), e1010834.
(16) Polyhach, Y.; Bordignon, E.; Jeschke, G. Rotamer libraries of spin labelled cysteines for protein studies. *Phys. Chem. Chem. Phys.* **2011**, *13* (6), 2356–2366.
